# Probabilistic projections of granular energy technology diffusion at subnational level

**DOI:** 10.1093/pnasnexus/pgad321

**Published:** 2023-09-29

**Authors:** Nik Zielonka, Xin Wen, Evelina Trutnevyte

**Affiliations:** Renewable Energy Systems, Institute for Environmental Sciences (ISE), Section of Earth and Environmental Sciences, University of Geneva, Geneva CH-1211, Switzerland; Renewable Energy Systems, Institute for Environmental Sciences (ISE), Section of Earth and Environmental Sciences, University of Geneva, Geneva CH-1211, Switzerland; Renewable Energy Systems, Institute for Environmental Sciences (ISE), Section of Earth and Environmental Sciences, University of Geneva, Geneva CH-1211, Switzerland

**Keywords:** probabilistic projections, spatial technology diffusion, granular energy technologies, model evaluation, climate change mitigation

## Abstract

Projections of granular energy technology diffusion can support decision-making on climate mitigation policies and infrastructure investments. However, such projections often do not account for uncertainties and have low spatial resolution. S-curve models of technology diffusion are widely used to project future installations, but the results of the different models can vary significantly. We propose a method to create probabilistic projections of granular energy technology diffusion at subnational level based on historical time series data and testing how various projection models perform in terms of accuracy and uncertainty to inform the choice of models. As a case study, we investigate the growth of solar photovoltaics, heat pumps, and battery electric vehicles at municipality level throughout Switzerland in 2000–2021 (testing) and until 2050 (projections). Consistently for all S-curve models and technologies, we find that the medians of the probabilistic projections anticipate the diffusion of the technologies more accurately than the respective deterministic projections. While accuracy and probabilistic density intervals of the models vary across technologies, municipalities, and years, Bertalanffy and two versions of the generalized Richards model estimate the future diffusion with higher accuracy and sharpness than logistic, Gompertz, and Bass models. The results also highlight that all models come with trade-offs and eventually a combination of models with weights is needed. Based on these weighted probabilistic projections, we show that, given the current dynamics of diffusion in solar photovoltaics, heat pumps, and battery electric vehicles in Switzerland, the net-zero emissions target would be missed by 2050 with high certainty.

**Significance Statement**

Most projections of energy technology diffusion, such as solar photovoltaics, heat pumps, and battery electric vehicles, are deterministic and hide future uncertainties. We provide a method that uses historical time series data of the diffusion of granular energy technologies to create probabilistic projections at subnational level. We find that there is no single best model of technology diffusion that outperforms the others in terms of accuracy and uncertainty. Thus, we demonstrate how multiple models could be weighted and combined. Projections from our approach inform policymakers not only about the future trends of granular technology diffusion but also about the likelihood of these trends.

## Introduction

Energy system models are widely used to quantify pathways that reach certain environmental or technological goals of the energy transition (1, 2). While energy optimization models mostly set normative techno-economic targets, these models hardly inform about the realistic pathways that are shaped by a broader socio-technical context (3, 4). In contrast to larger energy technologies, granular energy technologies with smaller, more modular capacities per unit, such as solar photovoltaics (PV), heat pumps, or battery electric vehicles (BEVs), show faster diffusion and learning and have more equitable access than less granular technologies (5). Realistic projections of granular energy technology diffusion would be useful to support decision-making on transition policies by contrasting the long-term target against the most likely expected pathway that the energy system is currently on (1, 6, 7). However, existing projections often have three major limitations. First, projections are often deterministic scenarios and do not account for uncertainties (8–10), thereby inducing overconfidence (9). If shown, uncertainties are often so broad that the projections become meaningless without information about what is more or less likely (11, 12). The quantification of uncertainties and likelihoods is particularly relevant when technology diffusion is nonlinear (13) or the projections directly assist decision-making (14, 15). Especially for granular energy technologies, their diffusion is difficult to foresee as it is dynamic and spatially scattered (16–18) and thereby subject to high variations and uncertainties. Second, projections often have a low spatial resolution (7, 19), which limits their relevance and use for local decision-makers that would need to take diverse assumptions to disaggregate national or other higher-level projections for own needs. Studies show that there are vast differences in how granular technologies like solar PV and heat pumps diffuse across subnational regions, calling for differentiated strategies in terms of local policies and local infrastructure (20–22). Third, projections mostly rely on a single model with numerous input assumptions (21, 23, 24), even if projections of different models can vary significantly (7, 23, 24) and can consequently cause overconfidence if the studies omit alternative models.

Past studies make efforts in adopting probabilistic approaches to account for uncertainties in technology projections (8, 10), e.g. with empirical methods (21, 25), Monte Carlo simulations (26–28), or by creating scenarios (29). We build on and extend this work by addressing the three abovementioned limitations at the same time. While Monte Carlo simulations are common to quantify uncertainties and sensitivities around energy scenarios (8, 30), modelers rarely use them to inform about the likelihoods of future developments. The key issue is that probability distributions of model input parameters often do not have a solid basis (10, 31). Expert elicitations can also provide estimates of uncertainties, but literature shows that elicitations can still notably deviate from reality (13, 31, 32) and that data-driven methods project future developments more accurately (33, 34).

To project future technology diffusion, studies commonly fit S-curve diffusion models to historical data (23, 35, 36). Standard S-curves describe the different phases of diffusion that technologies typically go through (37–39): initial formation followed by acceleration and exponential growth that increases up to an inflection point after which the growth eventually slows down until the curve saturates at its maximum. S-curves combine the influence of economic, social, and technological factors on technology diffusion over time (23, 37, 39) and show similar growth behavior as seen in history (28, 35, 36). Thereby, one can fit S-curves both at the late and early phases of technology diffusion to describe historical behavior or to project growth (23, 39). Standard S-curves are uniform as they have one inflection point. However, real diffusion can have multiple inflection points, for example, due to changes in technology policy, that can be modeled with bi-S-curves or curves of higher order (40–42). While most popular S-curve models are the uniform versions of logistic, Gompertz, and Bass models, there are plentiful alternative parametrizations of S-curves (23, 24, 43). The projections are sensitive to the choice of model parameters (36, 39, 44, 45) and can thus vary significantly across models (23, 24). Despite these observations, when creating projections of future technology diffusion, most studies nevertheless rely on one or two models (28, 37, 46), without providing an empirical and transparent examination of the suitability of the models to the investigated and out-of-sample data, let alone the quality of the projections. At the same time, fitting S-curves for technologies that are in their early phases of diffusion comes with high uncertainties (35, 37, 45). This is yet another call to add probabilities to projections of granular technologies that are in their early phases, to use multiple models, and to evaluate the performance of each model's projections over time.

We propose a method to create probabilistic projections of the diffusion of granular energy technologies at subnational level based on historical time series data and on testing which projection models perform best in terms of accuracy and uncertainty to inform the choice of the models. As a case study, we investigate the growth of three granular energy technologies of solar PV, heat pumps, and BEVs at municipality level throughout Switzerland historically in 2000–2021 (testing) and until 2050 (projection). Solar PV, heat pumps, and BEVs are key transition technologies to reduce Swiss greenhouse gas emissions (47–49). Modeling at the municipality level allows to identify and group similar diffusion patterns across municipalities (20–22) to create probabilistic density intervals while having as large as possible sample sizes, both to create these intervals and to test the performance of the projections (see Materials and methods). Historical time series can capture long-term trends by implicitly carrying relevant economic, social, or technological information related to the diffusion of technology (23, 50). Historical time series can also be used not only for fitting the models but also for testing them out of sample. We choose Switzerland because the uptake of granular technologies shows that groups of similar municipalities can be distinguished (20–22), because there are vast regional differences in policy contexts (51) and because data of high spatial resolution are available with open access. We analyze solar PV in installed capacity in three variables: absolute, per 100 inhabitants, and per technical potential in kW. We analyze the numbers of heat pumps and BEVs also in three variables: absolute, per 100 inhabitants, and per total number of existing buildings and civil passenger cars, respectively (see Materials and methods).

**Fig. 1.**
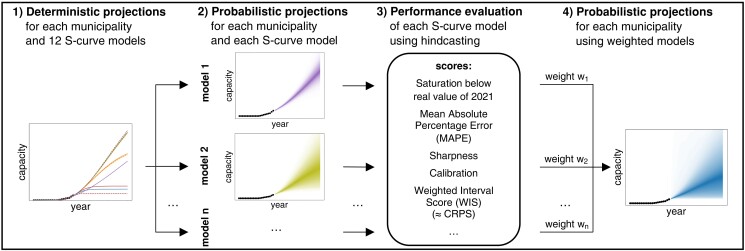
Methods flow chart for creating probabilistic projections of granular energy technology diffusion at subnational level. Appendix A, Section A.1 shows each step and the underlying assumptions in detail. In hindcasting, steps 1 and 2 are repeated, and, in each iteration, the historical time series data are split into training years and evaluation years. With each hindcasting iteration, one more year is used for fitting, respectively one less for evaluation, resulting in performance evaluation for 1- to 10-year-ahead projections. The WIS approximates the CRPS (52].

To create the probabilistic projections, we use a four-step process that we repeat for each of the 2,148 Swiss municipalities (Fig. 1; see Materials and methods). First, we fit 12 different S-curve models on the historical time series data of each technology's diffusion. As the fitting is individual for each S-curve, municipality, and technology, the projections can adapt to the current phases of technology diffusion, while the exact phase may be unknown beforehand. If the historical time series of technology in a municipality is quasi-static or highly fluctuating, we filter the time series out and create a projection for the municipality using mean values instead. Second, we combine the curves of municipalities with historically similar diffusion patterns to form a probabilistic density interval for each S-curve model and each municipality. Spatial interaction effects are not accounted for because they have been shown to be negligible among municipalities (21). Third, we evaluate each probabilistic projection using iterative hindcasting with out-of-sample testing and performance metrics. The metrics include, for instance, mean absolute percentage error (MAPE) and sharpness and calibration that sum to the weighted interval score (WIS) (52). The WIS approximates the continuous ranked probability score (CRPS) here (52). Finally, we convert the mean WIS of each projection model into weights that we use to combine the models to create a final probabilistic projection for each municipality. In doing so, we put higher weights on models that show comparatively reliable performance in out-of-sample hindcasting, even at early phases of technology diffusion. Altogether, the uncertainty in the projections comes from both the variability around curve fitting across municipalities (step 2) and different S-curve models (step 4). To showcase the capabilities of the probabilistic projections on a more prominent level than the one of each single municipality, we create national projections that are consistent with the projections at municipality level. To do so, we sum the quantiles of all Swiss municipalities. For instance, the national median means that all municipalities follow their median simultaneously, or, as the most extreme case, the national 99% quantile means that all municipalities follow their 99% quantile. We then compare the national probabilistic projection with published normative scenarios of the Swiss energy system with net-zero greenhouse gas emissions by 2050.

## Results

### Probabilistic vs. deterministic projections

**Fig. 2.**
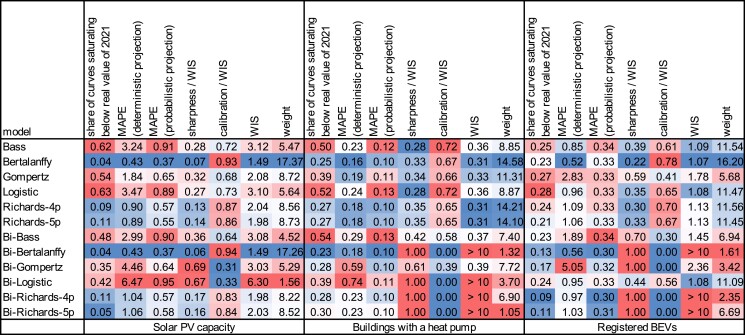
Heat map with weights and scores of model performance from hindcasting for solar PV capacity, heat pumps, and BEVs. For each column, colors rank each score from highest to lowest and vice versa for the weight. The shown values are means over all municipalities and hindcasting iterations with 1- to 10-year-ahead projections for solar PV and heat pumps and 1- to 4-year-ahead projections for BEVs. For temporal evolutions, see Figs. S10–S14. The MAPE of a probabilistic projection quantifies the error between the median value of the projection and the real value. To enhance comparability as some bi-S-curves have scores that are multiple orders higher than 10, the highest 2% of MAPE scores of the deterministic projections are removed for all models before taking the mean. For BEVs, the highest 2% of scores are also removed for WISs that approximate the CRPS. Models that still have mean scores above 10 are indicated.

Consistently for all S-curve models and technologies, we find in hindcasting that the medians of the probabilistic projections are more accurate than the respective deterministic projections (Fig. 2). The average MAPE over all municipalities and hindcasting iterations is consistently lower for the median of the probabilistic projections than for the deterministic projections and lowers even further with an increasing number of samples used in the creation of the probabilistic projections (Appendix A, Section A.2). For both types of projections, the magnitude of the MAPE depends on the technology and its historical time series. The projections of installed capacities of solar PV deviate more from the real diffusion than the respective projections of buildings with a heat pump and registered BEVs. The results are comparable to the ones for the diffusion per 100 inhabitants and per unit of technical potential (Appendix B, Section B.1). The difference in accuracy between the results of deterministic and probabilistic projections partially derives from the fact that the deterministic projections tend to underestimate and saturate at a lower level than the real diffusion (Fig. 2). As a comparison of solar PV, heat pumps, and BEVs points out, the behavior of low saturation appears in projections for both the near future and the comparatively distant future of up to 10 years.

When comparing the S-curve models, the hindcasting exercise reveals substantial differences in their performance in projecting future technology diffusion. While the probabilistic density intervals vary across technologies, municipalities, and years, Bertalanffy and both Richards models show on average lower MAPE and WIS than the ones of logistic, Gompertz, and Bass models (Fig. 2). Therefore, the estimated median values are both closer to the real diffusion of solar PV, heat pumps, and BEVs, and the probabilistic density intervals cover the distribution of the real diffusion more precisely. Consequently, the models of Bertalanffy and Richards receive higher weights for the final probabilistic projections in most cases (Fig. 2). However, the performance and thus the distribution of weights depend on the municipality and its historical time series of technology diffusion. For some municipalities, also the overall low-performing models of logistic, Gompertz, and Bass receive comparatively high weights of up to 55 (Appendix B, Section B.2). Note that for solar PV, the projections of the bi-S-curves of Bertalanffy and Richards show the same shape as the ones of their respective uniform S-curves in most municipalities, resulting in almost identical scores. For heat pumps and BEVs, the bi-S-curves mostly have different shapes and lower performance than the uniform models.

Beyond comparing the performance in terms of MAPE and WIS, alternative characteristics can play a role in choosing models for the projections. These characteristics can include the complexity of a model, the development of the historical time series of diffusion, or sharpness and distribution of the probabilistic density interval. The complexity of a model increases naturally with the number of parameters that can again increase the computation time for fitting the model. While the average time for curve fitting is lower than 0.1 s for our uniform S-curves, it increases up to 2 to 3 s for the bi-Richards curves. At the same time, the length and development of the historical time series of a diffusion influence the performance notably, so that especially the comparatively complex bi-S-curve models fail more often to provide practical probabilistic density intervals. The intervals of such models are so broad that the sharpness alone defines the WIS (Fig. 2). In contrast, for models with a low impact of sharpness on the WIS, the values of the real diffusion lay more toward the upper or lower end of the probabilistic density intervals. Consequently, the risk for real values to even lay outside the intervals is comparatively high, e.g. with the uniform models of Bertalanffy and Richards.

### Probabilistic projections for Switzerland

**Fig. 3.**
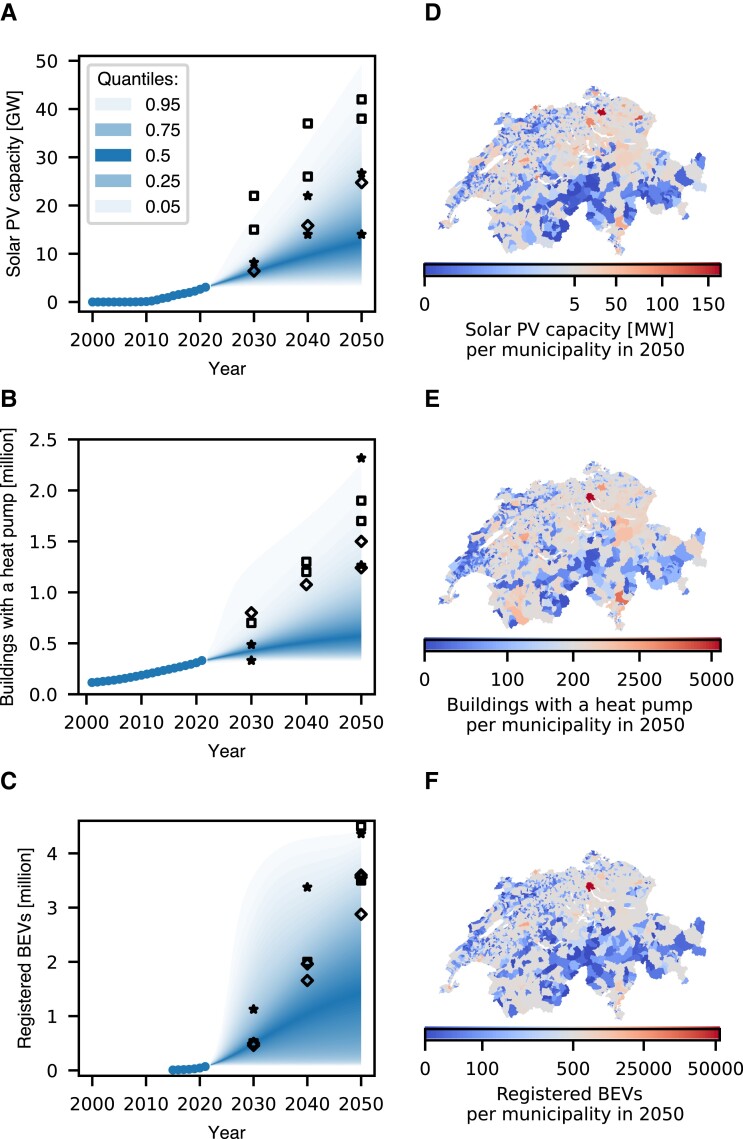
Probabilistic national projections a–c) of the diffusion of solar PV, heat pumps, and BEVs in Switzerland until 2050 and maps d–f) with the projected median values for each Swiss municipality in 2050, both with a quantile coloring scheme. The quantiles of the national projections are the sum of the respective quantiles of all municipalities. Markers set targets for reaching an energy system of net-zero greenhouse gas emissions by 2050, estimated in studies for the Swiss Federal Office of Energy (◊) (47) and (□) (53) and for the association of Swiss electricity companies (*) (48). If different scenarios exist, the highest and lowest values are shown.

According to our forward-looking national probabilistic projections for Switzerland with training on historical data until 2021, the diffusion of solar PV, heat pumps, and BEVs is unlikely to reach the levels that most scenarios from the literature estimate as needed for a Swiss energy system with net-zero greenhouse gas emissions in 2050 (Fig. 3a–c). The median of the projections of solar PV is at 12.5 GW in 2050, while most net-zero scenarios estimate total capacities that lay at the upper end of the probabilistic density interval, i.e. 25 GW (87% quantile) and higher. Only one study requires 14–15 GW (60% quantile) if Switzerland instead invests in wind power and natural gas, hydrogen, or other thermal power plants (48). For the number of buildings with a heat pump, the median projects a number of around 570,000 buildings in 2050, while all net-zero scenarios estimate numbers that are more than twice as high and lay above the 90% quantile, i.e. 1.2 million. Similar holds true for BEVs, where the projected median value of 1.4 million BEVs is only half of the lowest net-zero scenario for 2050, i.e. 79% quantile. However, the projections of BEVs for 2030 are in line with more scenarios from literature than the projections of solar PV and heat pumps and show a noticeably broader uncertainty range.

Across Switzerland, it can vary notably by how much various municipalities must increase their efforts to install and use solar PV, heat pumps, and BEVs. The subnational projections for 2050 estimate that higher capacities are generally concentrated close to population centers, i.e. in the northeast of Switzerland and around larger cities (Fig. 3d–f). Capacities are comparatively low in the south and in the northwest, which are both mountain regions of the Alps and Jura. This pattern is comparable to the diffusion in 2021 (Appendix B, Section B.3). Regional differences are most extreme for BEVs, where the number of BEVs in the municipality with the most BEVs is 200 times higher than the median of all municipalities in 2050. For solar PV and heat pumps, the respective factors are 54 and 30, respectively. However, the projections for the diffusion per 100 inhabitants (Appendix B, Section B.4) indicate that individual access to the capacities can still remain comparatively low in highly populated municipalities, e.g. around Zurich, Geneva, or Basel. Similar holds true for the shares of capacities over their technical potential (Appendix B, Section B.4). Potentials remain largely unexploited: only when the Swiss municipalities follow the 99% quantile path, 25% of municipalities might install more than 90% of their solar PV potential by 2050. For heat pumps, the respective shares are 36% of all municipalities and for BEVs, 78%. Interested readers can find the projections for each municipality and technology open source on Zenodo (54), annual updates (54) and a graphical example of probabilistic projections with estimated targets in Appendix B, Section B.5.

## Discussion

With the examples of the three granular technologies of solar PV, heat pumps, and BEVs, we show that our probabilistic projections provide both more accurate and reliable results than the respective deterministic projections and, at the same time, provide a more complete picture of the uncertainty. Not only is the MAPE of the probabilistic projections smaller, but also does the use of probabilistic density intervals and weighting of models compensate for problems that different projections of particular S-curve models can come with, e.g. underestimation, low saturation, or exceptionally broad density intervals. While the extent can vary by which the medians of the probabilistic projections outperform the deterministic projections and might differ for other case studies than Switzerland, it remains speculative which characteristics of the historical time series influence the performance of the projections to what extent. Our results plainly highlight that clear differences in the performance on average exist and that modelers should therefore in most cases project future diffusion of technologies together with probabilistic density intervals.

With our investigated models and evaluation criteria, we find that combining different models into one weighted probabilistic projection reduces trade-offs between advantages and disadvantages of single models. Especially the examples of Bertalanffy and some bi-S-curves highlight that projections of a model might be accurate while their probabilistic density intervals are either too narrow, i.e. having a low sharpness penalty in the WIS, or too broad to be meaningful, i.e. having a high sharpness penalty. Assigning weights to the models can take such differences into account while it passes on uncertainties that are latent in the selection of models. The weighting must hereby be individual for each technology and its historical time series in a municipality. Although the models of Bertalanffy and Richards most often receive the highest weights, their weights can vary across the technologies, municipalities, and years in hindcasting. Therefore, modelers should always consider multiple models when creating projections of technology diffusion and test them by means of hindcasting.

However, the use of particular S-curve models and the determination of weights come with high computational costs that can be out of proportion to the outcome. A model with more parameters might not eventually lead to more accurate projections. Examples of such are the four- and five-parameter Richards models that often create similar projections, while the five-parameter Richards needs more time to fit on the historical time series. The same is true for the bi-Bertalanffy or other bi-S-curve models that coincide with their respective uniform models that are faster to fit. The determination of weights takes time as hindcasting requires multiple repetitions of both curve fitting and creating and evaluating probabilistic density intervals. Only if the fitted curves of different models are distinct, they might justify the computation of projections from different models. Nevertheless, since it can be difficult to predict whether the projections of different models are distinct for a particular technology or municipality, the use of multiple models might be inevitable. Eventually, this uncertainty calls again for probabilistic density intervals in projections of technology diffusion.

The results and design of our approach have direct implications on how decision-makers on policies and infrastructure investments can use and interpret the probabilistic projections. First and foremost, the probabilistic approach provides information not only about the future trends of granular technology diffusion but also about the likelihood of these trends. Two common methodological shortcomings are overcome this way: overconfidence (9) and lack of meaning if probabilistic density intervals are too broad or show no likelihoods (11, 12). Second, our projections reflect uncertainties in a way that depends on the quality and length of the historical time series of diffusion, the variability around curve fitting across municipalities, and parametrization and historical performance of the different investigated models. Even if we include bi-S-curve models to account for the change in trends after, e.g. new policy, our approach for now provides only projections based on current diffusion dynamics. Hence, the approach cannot answer how future policies or context events, like subsidies or supply shortages, might accelerate or slow the projected diffusion. In line with Kaack et al. (25) and Morgan and Keith (9), combining probabilistic projections with scenarios might help to further illustrate technology diffusion and its uncertainties. Third, as the quantile of a national projection is subject to the condition that all municipalities follow this quantile simultaneously, our national projections do not represent scenarios in which some municipalities perform higher or lower than the quantile. Mathematically, there are almost 99 to the power of 2,148 scenarios possible (2,148 municipalities with 99 quantiles each), which are unpractical to calculate. Decision-makers may define specific scenarios of underperforming and overperforming municipalities before merging the projections of municipalities into a national projection. Having said that, we also find that our national projections based on aggregated municipal projections show at least the same or higher accuracy than projections from models fitted to national-level data (see Appendix A, Section A.7). At the same time, our modeling approach allows to create probabilistic projections that are consistent between municipality and national levels and are retrospectively tested in out of sample. The projections created with national data only cannot be disaggregated top-down without further assumptions and can only be deterministic (see Appendix A, Section A.7). Our results thereby encourage the collection, publication, and use of data at high spatial resolution so that both municipalities and higher-level jurisdictions can get their probabilistic projections of technology diffusion (e.g. Appendix B, Section B.5). Nevertheless, we acknowledge that the choice of spatial resolution depends foremost on the research questions.

In addition to decision-makers, the design of our methods has also direct implications on how modelers can use and interpret the probabilistic projections. Our methods rely on only a few input assumptions, which make the methods applicable specifically to cases where the availability of different types of data is limited. Nonetheless, our subnational projections rely on the availability of good-quality subnational data of high spatial resolution and for as long a historical time series as possible. Generally, our methods allow for lower spatial resolution than the municipality level or less granular technologies with little spatial distribution too. However, a lower resolution or distribution could reduce the number of entities with a similar diffusion pattern and thereby provide a lower sample size to create the probabilistic density intervals. Consequently, the use of data at lower resolution could reduce the quality and practicality of the intervals as, without out-of-sample hindcasting, these might become too broad or too poorly defined to be meaningful. The risk of poorly defined intervals is also high for technologies at early phases of their diffusion and their accordingly short historical time series, even if our methods are, due to their receptive design and weighting, applicable to time series data at any phase of technology diffusion. For instance, the density intervals of BEVs projections are comparatively broad, and a sixth of all municipalities are excluded from curve fitting (see Materials and methods). Consequently, modelers might have to adjust the way we create the intervals and use additional input parameters. Our approach of dealing with missing data by using average values from other regions (Appendix A, Section A.5) could be refined further using additional assumptions.

Altogether, our work builds on and extends the existing literature on granular energy technologies, projections, and uncertainties, while our method stands out in combining municipalities with similar diffusion patterns, multiple models, and hindcasting-based performance evaluation and weighting to create probabilistic projections. Drawing projections only from historical time series of diffusion is justified as the time series implicitly includes technological, socioeconomic, and political factors that drive the diffusion (23). Nevertheless, if additional data are available, the use of such factors might improve the projections (50, 55). At the same time, the direct use of alternative diffusion models, evaluation criteria, or types of weighting can influence the projections. Although we provide an analysis of multiple models and criteria, future work can investigate them further and analyze how the use of different criteria impacts the choice of models in different years of a technology diffusion or in different regions. Our Swiss case study encourages modelers to carefully compare and select models individually for each technology and region, as there can exist a notable variance in model performance. We expect such variance not only across Switzerland but also in other regions.

## Materials and methods

### Data

The data used in our study are publicly available at different spatial resolutions and for different time periods (56–58). For consistency, we aggregate all data to the 2,148 Swiss municipalities that existed at the end of 2021 (59, 60) and start the time series of technology diffusion in the first year of available data. Our data set of solar PV registers all installations from 2000 to 2021 that are in use and have a minimum capacity of 2 kW (56), covering 89% of the real existing total capacity (61). We use solar PV capacities that are attached to or integrated into buildings and make up around 96% of the available data set of solar PV in Switzerland (56). We approximate the number of heat pumps in 2001–2021 with the number of buildings that are registered in the Swiss Federal Register of Buildings and Dwellings (57) and heated by at least one heat pump as a primary or secondary heating system for space heating or warm water. See Appendix A, Section A.3 for a detailed derivation of the time series. The data set of BEVs records the numbers of all civil passenger cars registered at the post address of their owners in 2015–2021 (58). For analyzing diffusion per number of inhabitants, population sizes per municipality are available for the years 2000 and 2007–2020 (62), and we linearly interpolate them for 2001–2006 and use the numbers of 2020 also for 2021. For analyzing diffusion per unit of maximum technical potential, we use the technical potential of solar PV on currently existing roofs and facades (63) in kW with local capacity factors (64, 65); see Appendix A, Section A.4. For heat pumps and BEVs, we use the currently existing number of buildings (57) and civil passenger cars (58), respectively.

### Methods

Our four-step process to create a probabilistic projection consists of two main parts: the creation of deterministic projections and probabilistic projections for each S-curve model (steps 1 and 2 in Fig. 1) and the creation of a final projection that combines the probabilistic projections of the models (steps 3 and 4 in Fig. 1). Appendix A, Section A.1 shows each step and the underlying assumptions in detail. Prior to the four steps, we remove municipalities with missing, quasi-static, or highly fluctuating historical time series from our data set and use mean growth rates and model weights of all municipalities for the projections (Appendix A, Section A.5). We exclude municipalities with historical time series in which one of the last three values is 0, the last 5 (for BEVs: 3) values are the same, or the values drop by half or more from one year to another. Correspondingly, we remove for solar PV, heat pumps, and BEVs 2.5%, 3.5%, and 16.6% of municipalities, respectively.

In the first part, we fit 12 different S-curve models on the historical time series of the diffusion of a technology to create 12 deterministic projections. The curve fitting uses differential evolution and nonlinear least squares optimization to minimize the residuals of a curve to the given points of the time series to find the optimal set of parameters of a curve. For details, see Appendix A, Section A.6. Then, we create one probabilistic projection for each municipality and each S-curve model by combining all deterministic projections of similar municipalities for each model and calculating the quantiles of the resulting distribution. Before combining the considered municipality with another one, we normalize the deterministic projections using the value of the last year used for curve fitting and afterward multiply the quantiles with the last value of the historical time series of the considered municipality. We consider two municipalities similar if the mean Euclidean distance between their normalized historical time series is lower than the 30% quantile of the mean Euclidean distances to all municipalities. See Appendix A, Section A.2 for sensitivity analysis and discussion of the chosen 30% quantile. The normalized deterministic projections of the municipalities already provide a range for the probabilistic density intervals that alternative sampling would need to compute in addition. By using historical time series only, the grouping of similar municipalities is purely data driven and leaves out any other geographical characteristics like proximity or socioeconomic variables and regulations that are spatially diffuse in Switzerland (20–22). However, we acknowledge that alternative multivariable and nondata-driven grouping with additional assumptions is theoretically possible and could lead to alternative density intervals.

In the second part, we evaluate the performance of each probabilistic projection using iterative hindcasting. For this, we repeat steps 1 and 2 and split the historical time series data into training years and evaluation years. With each hindcasting iteration, we use one more year for fitting and respectively one year less for evaluation. In each iteration, we calculate metrics of model performance relative to each observation for 1- to 10-year-ahead projections of solar PV and heat pumps, and 1- to 4-year-ahead projections of BEVs. The metrics include the following:

- The share of curves saturating below the time series value of 2021;
- MAPE to take the different scales of technology diffusion across the municipalities into account; and
- WIS as the sum of sharpness and calibration and with the use of interval weights that approximate the WIS to the percentage version of the CRPS (52, 66).

Based on the performance of the 12 S-curve models, we assign weights that are individual for each municipality and use the weights to combine the probabilistic projections. We derive the calculation of weights from methods of ensemble weather forecasting where the use of inverse error variance outperforms equal weighting (67, 68). The mean squared WIS acts as the error variance in our case.

### S-curves

The S-curve models we investigate are listed below and comprise 6 uniform models and linear combinations of each uniform model with itself, creating six bi-S-curves to model two growth phases, e.g. due to change in policies. We investigate two common symmetric S-curves, i.e. logistic and Bass, and four asymmetric curves, i.e. Gompertz, Bertalanffy, a four-parameter version, and a five-parameter version of the generalized Richards model. The function value *f*(*t*) describes the installed capacity or number of solar PV, heat pumps, or BEVs in their specific units, e.g. kW, in year *t*. We add a vertical shift *z* so that the curves can handle time series that begin with nonzero values. (*C* − *z*) is the level of saturation and both *C* and *z* have the same unit as *f*(*t*). The time shift *t*_0_ is given in years, while *p*, *q*, *k*, *d*, and *b* are unitless curve parameters that are specific to each curve. See Appendix A, Section B.6 for parameter limits.

Bass [adapted from Bass 69):


$$f(t)=(C-z)\cdot \frac{1-\exp\;(-(p+q)(t-t_{0}))}{1+\frac{q}{p}\cdot \exp\;(-(p+q)(t-t_{0}))}+z.$$ (1)

Bertalanffy [adapted from Höök et al. 23):


$$f(t)=(C-z)\cdot (1-b\cdot \exp\;(-k\cdot (t-t_{0})){)}^{3}+z.$$ (2)

Gompertz [adapted from Tjørve and Tjørve and Gompertz 43, 70):


$$f(t)=(C-z)\cdot \exp\;(-\exp\;(-k\cdot (t-t_{0})))+z.$$ (3)

Logistic [adapted from Meyer 42):


$$f(t)=\frac{C-z}{1+\exp(-k\cdot (t-t_{0}))}+z.$$ (4)

Richards-4p [adapted from Tjørve and Tjørve 43):


$$f(t)=(C-z)\cdot {\left(1-\frac{1}{d}\cdot \exp\;(-k\cdot (t-t_{0}))\right)}^{d}+z.$$ (5)

Richards-5p [adapted from Höök et al. 23):


$$f(t)=(C-z)\cdot (1-b\cdot \exp\;(-k\cdot (t-t_{0})){)}^{d}+z.$$ (6)

### Comparison with net-zero scenarios

To compare the probabilistic projections of the diffusion of solar PV, heat pumps, and BEVs in Switzerland, we add a set of target values from studies that model a Swiss energy system reaching net-zero greenhouse gas emissions by 2050 (Fig. 3). If a study provides different scenarios, we add the highest and lowest values. Two studies for the Swiss Federal Office of Energy (47, 53) provide targets that complement the measures of the Swiss government to decarbonize the Swiss energy system (71). The scenarios of the studies model different shares of electrification, heating networks, and biofuels and their implications on the power grid. The association of Swiss electricity companies reports scenarios that analyze the level of integration of Switzerland in the European energy market and different rates of infrastructure investments (48). For solar PV, we convert annual generation levels into capacities using the national average of local capacity factors at 0.155 (64, 65). For heat pumps, we convert the increase in heat supply using the heat supply of the reference years of the studies and the number of buildings in the Swiss Federal Register of Buildings and Dwellings (57). For BEVs, we multiply shares with the total number of civil passenger cars in 2021 (58).

## Supplementary Material

pgad321_Supplementary_Data

## Data Availability

The probabilistic projections of solar PV, heat pumps, and BEVs for all 2,148 Swiss municipalities are provided on Zenodo (54) and get updated anually. The historic time series data are publicly available at Swiss Federal Office of Energy (56) and Federal Statistical Office (57, 58).
