# Supplementary material for: Probabilistic projections of granular energy technology diffusion at subnational level
Source: PNAS Nexus. 2023 Sep 29;2(10):pgad321. doi: 10.1093/pnasnexus/pgad321 (PMC10578461; doi:10.1093/pnasnexus/pgad321)
Supplement: pgad321_Supplementary_Data [file pgad321_supplementary_data.pdf]

Supplementary material:
Probabilistic projections of granular energy technology diffusion at subnational level

PNAS Nexus, pgad321, https://doi.org/10.1093/pnasnexus/pgad321

Authors: **Nik Zielonka[1]*, Xin Wen[1], Evelina Trutnevyte[1]**

[1] Renewable Energy Systems, Institute for Environmental Sciences (ISE), Section of Earth and Environmental Sciences, University of Geneva, Switzerland

* corresponding author (University of Geneva, Boulevard Carl Vogt 66, CH-1211 Geneva 4, Switzerland; +41 22 379 08 26; Nik.Zielonka@unige.ch)

Table of Contents

Appendix A......2

A.1 Flow chart of methods and assumptions......2

A.2 Sensitivity analysis of the influence of the quantile of the mean Euclidean distance on the probabilistic projections......6

A.3 Data on heat pumps......8

A.4 Data on local capacity factors for solar PV and technical potential......9

A.5 Projections for municipalities with missing, quasi-static or highly fluctuating historical time series of diffusion......10

A.6 Determination of S-curve parameters......10

A.7 Comparison of national projections from modeling on national data and from aggregating municipality-level probabilistic projections......11

Appendix B......13

B.1 Heat maps with weights and scores of model performance from hindcasting......13

B.2 Distribution of weights for probabilistic projections of solar PV, heat pumps, and BEVs......15

B.3 Diffusion of solar PV, heat pumps, and BEVs across Switzerland in 2021......16

B.4 Diffusion of solar PV, heat pumps, and BEVs across Switzerland in 2050......17

B.5 Probabilistic projections of the diffusion of solar PV and BEVs in a municipality......18

B.6 Temporal evolution of the mean absolute percentage error for probabilistic and deterministic projections of solar PV, heat pumps, and BEVs......19

B.7 Temporal evolution of sharpness, calibration, and weighted interval score for probabilistic and deterministic projections of solar PV, heat pumps, and BEVs......21

References......24

Appendix A contains supplementary descriptions and discussions on the data and methods that we use in our study. Appendix B shows additional results of our case study.

Appendix A

A.1 Flow chart of methods and assumptions

Our main article visualizes the creation of our probabilistic projections in a four-step process. To ensure transparency, Table S1a-e lists the input data, a short description of tasks performed, the underlying assumptions, and the output that is created. We illustrate the steps using our case study on solar photovoltaics (PV), heat pumps, and battery electric vehicles (BEVs) in Swiss municipalities.

Table S1a. Step 0 of the methods flow for creating probabilistic projections of technology diffusion.

**0. Data preparation**		
Input	Description	Output
Raw data:<br>- Historical time series of a diffusion of a technology<br>- Limits of diffusion (e.g. technical potentials)<br>- List of municipalities<br>- Size of population for each municipality	Task:<br>Clean, filter and merge raw data.<br><br>Assumptions on historical data:<br>- Solar PV: total installed capacity in the installation year.<br>- Heat pumps: approximated by the number of registered buildings with a heat pump and an installation year based on the combination of the construction year of the building and the year when the heating system data was updated (see Section A.3).<br>- Battery electric vehicles (BEVs): number of registered BEVs in the municipality of the owner's address.<br><br>Assumptions on potentials:<br>- Solar PV: total installable capacity on roofs and facades (technical potential)<br>- Heat pumps: total number of registered buildings in 2021<br>- BEVs: total number of registered civil passenger cars in 2021	Historical time series of a diffusion of a technology:<br>- Solar PV (years 2000-2021): installed capacity (absolute, per 100 inhabitants, and per technical potential in kW)<br>- Heat pumps (2001-2021): number of buildings with a heat pump (absolute, per 100 inhabitants, and per total number of registered buildings in 2021)<br>- BEVs (2015-2021): number of registered civil passenger cars (absolute, per 100 inhabitants, and per total number of registered civil passenger cars in 2021)

Table S1b. Step 1 of the methods to create probabilistic projections of technology diffusion.

1. **Deterministic projections for each municipality and 12 S-curve models**		
Input	Description	Output
Historical time series of a diffusion of a technology<br><br>Six uniform S-curve models:<br>- Bass<br>- Bertalanffy<br>- Gompertz<br>- Logistic<br>- Four-parameter Richards (Richards-4p)<br>- Five-parameter Richards (Richards-5p)<br><br>Six bi-S-curve models:<br>- Bi-Bass<br>- Bi-Bertalanffy<br>- Bi-Gompertz<br>- Bi-Logistic<br>- Bi-Richards-4p<br>- Bi-Richards-5p	Task:<br>1. Exclude historical time series with missing, static, quasi-static and highly fluctuating values.<br><br>2. Fit all S-curve models to the historical time series of a diffusion of a technology for a given range of years using non-linear least squares optimization:<br>- Solar PV: 2000-2021<br>- Heat pumps: 2001-2021<br>- BEVs: 2015-2021<br>A differential evolution method determines the initial guess of model parameters used in the least squares optimization.<br><br>Assumptions:<br>- The diffusion of a technology follows the shape of an S-curve.<br>- The range of possible parameter values is bounded.	Deterministic projections for each technology, each municipality and twelve S-curve models

Table S1c. Step 2 of the methods to create probabilistic projections of technology diffusion.

**2. Probabilistic projections for each municipality and each S-curve model**		
Input	Description	Output
Historical time series of a diffusion of a technology<br><br>Deterministic projections for each technology, each municipality and twelve S-curve models	Task:<br>1. Define similar municipalities: Normalize using the value of the last year used for curve fitting and compare the historical time series by calculating the Euclidean distance.<br><br>2. Create probabilistic projections: Combine all deterministic projections of similar municipalities for each S-curve model and calculate the quantiles of the resulting distribution.<br><br>Assumptions:<br>- Municipalities are considered similar if the mean Euclidean distance between their normalized historical time series is lower than the 30% quantile of the mean Euclidean distance to all municipalities (see Section A.2).<br>- Probabilistic density intervals derive from the variation of projections of different S-curve models and similar municipalities.	Probabilistic projections for each technology, each municipality and twelve S-curve models

Table S1d. Step 3 of the methods to create probabilistic projections of technology diffusion.

**3. Performance evaluation of each S-curve model using hindcasting**		
Input	Description	Output
Probabilistic projections for each technology, each municipality and twelve S-curve models<br><br>Metrics of model performance:<br>- Saturation below real value of 2021<br>- Mean absolute percentage error (MAPE)<br>- Sharpness<br>- Calibration<br>- Weighted interval score (WIS)	Task:<br>1. Evaluate the performance of each probabilistic projection using iterative hindcasting: Repeat steps 1 and 2, split the historical time series data and vary the years used for curve fitting and evaluation in each iteration, and calculate metrics of model performance relative to each observation for<br>- 1- to 10-year-ahead projections (solar PV and heat pumps)<br>- 1- to 4-year-ahead projections (BEVs)<br>2. Assign weights to each probabilistic projection of an S-curve model and municipality using the inverse of the mean squared weighted interval score.	Scores of performance metrics of deterministic and probabilistic projections for each S-curve model, municipality, and technology<br><br>Weights for each S-curve model of each municipality and technology

Table S1e. Step 4 of the methods to create probabilistic projections of technology diffusion.

**4. Probabilistic projections for each municipality using weighted models**		
Input	Description	Output
Probabilistic projections for each technology, each municipality and twelve S-curve models<br><br>Weights for each S-curve model of each municipality and technology	Task:<br>1. Create probabilistic projections by combining the probabilistic projections the S-curve models according to the calculated weights and taking the quantiles of the resulting distribution.<br>2. Create probabilistic projections for municipalities that have been excluded in step 1 using average weights and growth rates.<br><br>Assumptions:<br>- The best performing models of the past will also be the best in the future.<br>- The diffusion of technologies in excluded municipalities will follow the shape of S-curves from the first projected year onwards.	Probabilistic projections for each municipality and technology based on weighted models

A.2 Sensitivity analysis of the influence of the quantile of the mean Euclidean distance on the probabilistic projections

To justify the use of the 30% quantile of the mean Euclidean distance as a cutoff to define whether two municipalities are similar, we perform a sensitivity analysis of the quantile value and discuss the tradeoffs coming with the choice of a value. We perform the sensitivity analysis on the case of solar PV capacity since the technology both has the longest historical time series available for hindcasting and results in largest differences in performances of the models compared to the other technologies. We find that the performance of all models in terms of mean absolute percentage error (MAPE) and weighted interval score (WIS) consistently increases or decreases with the increase or decrease of the quantile cutoff value and by this, the number of curves based on which the probabilistic density intervals are created (Figure S1 and Figure S2). Here, the comparatively low performing models show highest sensitivity to the used quantile, i.e. the magnitudes of increase or decrease in the performance are highest. Consequently, the difference in weights and scores between the models increases with the use of a lower quantile and decreases with the use of a higher quantile.

Although the results of the sensitivity analysis point towards using a higher quantile, there are a couple of tradeoffs that come with a higher cutoff: (i) computational costs increase, especially in terms of computation time and required memory storage, (ii) the degree of

similarity of additional historical time series treated as similar in the creation of probabilistic projections lowers, and by this, (iii) the shape of the probabilistic density intervals of all municipalities become more similar to each other and thus counteract the goal of creating individual projections for each municipality. The value of the quantile also should not be too low since a low quantile can result in a number of curves that might not be too low to create a meaningful probabilistic density interval that can compensate outliers in the set of curves. As we calculate 99 quantiles that make up the probabilistic density interval (0.01-0.99), we target to have a couple of hundred curves based on which we create the probabilistic density intervals for each municipality. Taking all tradeoffs into consideration, we use the 30% quantile as a compromise for the computation of the results of our case study.

model	MAPE	sharpness / WIS	calibration / WIS	WIS	weight	MAPE	sharpness / WIS	calibration / WIS	WIS	weight	MAPE	sharpness / WIS	calibration / WIS	WIS	weight	MAPE	sharpness / WIS	calibration / WIS	WIS	weight	MAPE	sharpness / WIS	calibration / WIS	WIS	weight
Bass	1.04	0.23	0.77	3.57	4.83	0.97	0.26	0.74	3.31	5.16	0.91	0.28	0.72	3.12	5.47	0.80	0.36	0.64	2.58	5.87	0.65	0.44	0.56	2.17	6.46
Bertalanffy	0.37	0.06	0.94	1.52	18.64	0.37	0.06	0.94	1.51	17.99	0.37	0.07	0.93	1.49	17.37	0.37	0.07	0.93	1.48	16.62	0.36	0.07	0.93	1.46	15.58
Gompertz	0.77	0.25	0.75	2.57	7.90	0.71	0.28	0.72	2.33	8.36	0.65	0.32	0.68	2.08	8.72	0.56	0.38	0.62	1.79	9.29	0.46	0.42	0.58	1.57	9.85
Logistic	1.02	0.23	0.77	3.55	4.97	0.96	0.26	0.74	3.28	5.30	0.89	0.27	0.73	3.10	5.64	0.78	0.35	0.65	2.57	6.07	0.63	0.44	0.56	2.12	6.76
Richards-4p	0.58	0.11	0.89	2.14	8.67	0.57	0.12	0.88	2.09	8.61	0.57	0.13	0.87	2.04	8.56	0.56	0.14	0.86	2.00	8.52	0.55	0.15	0.85	1.95	8.48
Richards-5p	0.57	0.12	0.88	2.08	8.78	0.56	0.13	0.87	2.03	8.76	0.55	0.14	0.86	1.98	8.73	0.55	0.15	0.85	1.93	8.69	0.54	0.16	0.84	1.88	8.67
Bi-Bass	1.03	0.29	0.71	3.54	4.31	0.97	0.32	0.68	3.31	4.41	0.90	0.36	0.64	3.08	4.52	0.83	0.44	0.56	2.69	4.65	0.74	0.52	0.48	2.39	4.78
Bi-Bertalanffy	0.37	0.06	0.94	1.52	18.51	0.37	0.06	0.94	1.51	17.87	0.37	0.06	0.94	1.49	17.26	0.37	0.07	0.93	1.48	16.50	0.36	0.07	0.93	1.47	15.46
Bi-Gompertz	0.73	0.64	0.36	3.28	5.13	0.68	0.66	0.34	3.16	5.22	0.64	0.69	0.31	3.03	5.29	0.56	0.73	0.27	2.84	5.31	0.47	0.77	0.23	2.67	5.23
Bi-Logistic	1.09	0.67	0.33	7.73	1.41	1.02	0.67	0.33	6.90	1.49	0.95	0.67	0.33	6.30	1.56	0.86	0.71	0.29	5.46	1.69	0.71	0.78	0.22	4.86	1.82
Bi-Richards-4p	0.59	0.15	0.85	2.09	8.27	0.58	0.16	0.84	2.03	8.25	0.57	0.17	0.83	1.98	8.22	0.56	0.18	0.82	1.93	8.18	0.55	0.20	0.80	1.87	8.22
Bi-Richards-5p	0.59	0.14	0.86	2.14	8.47	0.58	0.15	0.85	2.08	8.47	0.58	0.16	0.84	2.03	8.52	0.57	0.17	0.83	1.97	8.56	0.55	0.19	0.81	1.91	8.61
	quantile: 20%					quantile: 25%					quantile: 30%					quantile: 35%					quantile: 40%				

Figure S1. Heat map with weights and scores of model performance from hindcasting for solar PV capacity for different quantiles used as a similarity criterion in the creation of probabilistic projections. The values for the 30% quantile are the same as in the heat map in the main article (Figure 2). All shown values are means over all municipalities and hindcasting iterations with 1- to 10-year ahead projections. The mean absolute percentage error (MAPE) of a probabilistic projection quantifies the error between the median value of the projection and the real value. For each column, colors rank each score from highest to lowest and vice versa for the weight. WIS: Weighted interval score that approximates the continuous ranked probability score.

model	MAPE	sharpness / WIS	calibration / WIS	WIS	weight	MAPE	sharpness / WIS	calibration / WIS	WIS	weight		MAPE	sharpness / WIS	calibration / WIS	WIS	weight	MAPE	sharpness / WIS	calibration / WIS	WIS	weight
Bass	0.14	-0.17	0.07	0.15	-0.12	0.07	-0.08	0.03	0.06	-0.06		-0.12	0.28	-0.11	-0.17	0.07	-0.29	0.59	-0.23	-0.30	0.18
Bertalanffy	0.01	-0.09	0.01	0.02	0.07	0.00	-0.04	0.00	0.01	0.04		-0.01	0.05	0.00	-0.01	-0.04	-0.01	0.09	-0.01	-0.02	-0.10
Gompertz	0.19	-0.23	0.11	0.23	-0.09	0.10	-0.12	0.06	0.12	-0.04		-0.14	0.20	-0.09	-0.14	0.06	-0.29	0.33	-0.15	-0.25	0.13
Logistic	0.14	-0.16	0.06	0.15	-0.12	0.07	-0.07	0.02	0.06	-0.06		-0.12	0.27	-0.10	-0.17	0.08	-0.30	0.59	-0.22	-0.31	0.20
Richards-4p	0.02	-0.14	0.02	0.05	0.01	0.01	-0.07	0.01	0.02	0.01		-0.01	0.07	-0.01	-0.02	-0.01	-0.02	0.15	-0.02	-0.05	-0.01
Richards-5p	0.02	-0.15	0.02	0.05	0.01	0.01	-0.08	0.01	0.02	0.00		-0.01	0.08	-0.01	-0.02	-0.01	-0.03	0.16	-0.03	-0.05	-0.01
Bi-Bass	0.14	-0.20	0.11	0.15	-0.05	0.07	-0.10	0.06	0.07	-0.02		-0.08	0.21	-0.12	-0.13	0.03	-0.18	0.44	-0.25	-0.22	0.06
Bi-Bertalanffy	0.01	-0.09	0.01	0.02	0.07	0.00	-0.04	0.00	0.01	0.04		-0.01	0.05	0.00	-0.01	-0.04	-0.01	0.09	-0.01	-0.02	-0.10
Bi-Gompertz	0.15	-0.07	0.15	0.08	-0.03	0.08	-0.04	0.08	0.04	-0.01		-0.12	0.06	-0.14	-0.06	0.00	-0.26	0.11	-0.25	-0.12	-0.01
Bi-Logistic	0.14	0.00	0.01	0.23	-0.10	0.07	0.00	0.00	0.09	-0.05		-0.10	0.07	-0.14	-0.13	0.08	-0.25	0.16	-0.33	-0.23	0.17
Bi-Richards-4p	0.03	-0.15	0.03	0.05	0.01	0.01	-0.07	0.01	0.02	0.00		-0.01	0.08	-0.02	-0.03	-0.01	-0.04	0.16	-0.03	-0.06	0.00
Bi-Richards-5p	0.03	-0.15	0.03	0.06	-0.01	0.01	-0.08	0.01	0.03	-0.01		-0.02	0.07	-0.01	-0.03	0.00	-0.04	0.16	-0.03	-0.06	0.01
	quantile: 20%					quantile: 25%					30%	quantile: 35%					quantile: 40%				

Figure S2. Heat map with percentage differences of weights and scores of model performance for solar PV capacity for different quantiles used as a similarity criterion in the creation of probabilistic projections compared to weights and scores of the 30% quantile. Figure S1 shows the absolute weights and scores. Colors rank each value from zero (white) to highest and lowest difference (red). WIS: Weighted interval score that approximates the continuous ranked probability score.

A.3 Data on heat pumps

The Swiss Federal Register of Buildings and Dwellings (1) registers for each building the heating technology installed as a primary or secondary heating system for space heating or warm water. However, the register does not specify the installed capacities, years of installation, nor is it clear how complete the register is in terms of total number of registered buildings and up-to-date information on heating systems. Nevertheless, it is the most complete dataset of buildings in Switzerland that, for instance, the Federal Statistical Office (2–4) or Energy Reporter (5) use for aggregated statistics. We take the following steps to derive a historical time series of the diffusion of heat pumps in Switzerland:

1. We filter for each municipality the register for existing buildings that have a heat pump registered as primary or secondary heating system for space heating or warm water. It is indistinguishable whether a building uses a heating system for multiple purposes or whether there exist separate heat pumps for the different heating purposes.
2. We assume that the installation year of a heat pump in a building is the same as the construction year of the building. If the construction year is missing in the register, we use the year in which the information of the primary space heating system is updated in the register. Note that the date of information update is not necessarily the same as the installation year. If the date of information update of the primary space heating system is missing, we use the earliest year of the three dates of information update of secondary space heating system, primary warm water heating system, and secondary warm water heating system. We argue that the error that derives from our assumption to use the construction

year of a building is limited since the average lifetime of a heating system is 20 years (6) and the maximum number of years we use for curve fitting is 21. Therefore, we expect the real installation year to lay within the time range that we use for curve fitting.

3. Since the first two steps can result in multiple remaining entries of the same building, e.g. if a heat pump is registered as a heating system for space heating and warm water, we remove all duplicates to avoid double counting. For practical reasons, we count buildings only more than once if the installation years that we assume are different for primary or secondary space heating or warm water supply. This is the case for less than 0.7% of all registered buildings with heat pumps.

When we compare the sum of our derived historical time series with other datasets, we see comparable diffusion of heat pumps. For instance, the model for the electrical heat pump statistics (7, 8) estimates a similar level of diffusion in Switzerland, although its growth rate is higher. As both their model and our derivation use assumptions, it remains uncertain how the real diffusion evolves over time. However, we assume that the errors spread equally across the municipalities and therefore have only little effect on the comparison of different municipalities.

A.4 Data on local capacity factors for solar PV and technical potential

We use local capacity factors to estimate the average annual power generation of installed solar PV capacities in each municipality of Switzerland and to convert generation potentials into capacities. First, we download the capacity factors for solar PV from Renewables.ninja (9, 10) for all coordinates of the geographical centers of the 2’148 Swiss municipalities (11). We take the “MERRA2” dataset and extract the capacity factors for every hour of the latest available year, i.e. 2020. For every municipality, we assume a system loss of 0.1, no tracking, a tilt of 35° and an azimuth of 180° to represent the average orientation angles of solar PV panels in a municipality. Second, we take the annual solar power generation potentials of each municipality estimated in a scenario that considers solar PV on roofs and facades (12). Finally, we convert the generation potentials into capacities using the local capacity factors and an average electricity output ratio of 950 kWh/kW for Switzerland that represents a conservative estimate based on electricity outputs of solar PV panels observed per year (13, 14). We calculate electricity output ratios for each municipality by weighting the Swiss average electricity output with the annual mean of a local capacity factor over the annual mean of the average capacity factor of all municipalities.

A.5 Projections for municipalities with missing, quasi-static or highly fluctuating historical time series of diffusion

The diffusion of technologies in their early phases and thereby the fitting of S-curves is subject to high uncertainties (15–17). The S-curve models we use in our study assume growth in technology diffusion that the historical time series data might not represent if the time series is static, quasi-static or highly fluctuating. Therefore, we exclude municipalities prior to applying our four-step methods if their historical time series meet at least one of the following criteria:

- All values are zero;
- One of the last three values used for curve fitting is zero;
- The last five (for BEVs: three) values used for curve fitting are the same;
- The historical time series drops by at least 50% in value from one year to another.

One example of outliers is the municipality of Dielsdorf where the number of registered BEVs more than doubles from 2016 to 2018 and eventually drops to almost half of the vale of 2015 in 2021. We assume that this atypical behavior is due to registrations of the car manufacturer Bayerische Motoren Werke that has its Swiss headquarters in Dielsdorf (18) and the time series does not represent the true diffusion of BEVs in this municipality.

To create a probabilistic projection of technology growth for the excluded municipalities, we assume that their technology diffusion follows the average growth of all Swiss municipalities from the first projected year, i.e. 2022, onwards. First, we take for each S-curve model the quantiles of the combined normed probabilistic projections of all non-excluded Swiss municipalities. Second, we calculate the average weights of the S-curve models of all non-excluded Swiss municipalities (see, e.g. Figure S4). Finally, we multiply the quantiles with the last value of the historical time series of an excluded municipality to create a probabilistic projection and combine the projections using the average model weights. If the last value in the historical time series is zero, we multiply with a dummy variable that we subtract again from the projected values, to shift the starting point of the diffusion back to zero. We define the dummy variable as the median of the initial installation capacity of all municipalities. The initial installation capacity is the total installed capacity of a municipality in the first year in which the capacity is larger than zero.

A.6 Determination of S-curve parameters

To determine the values of S-curve parameters, as shown in *Materials and methods,* so that the curve fits the historical time series of a diffusion of a technology best, we use a non-linear least squares optimization with initial guess and bounds for the parameters. Since the

initial guess of model parameters influences the determination of optimal parameters notably, we employ a differential evolution method (19, 20) that uses random inputs to find the optimal set of values for the initial guess. For each S-curve model, we feed the same parameter bounds into the differential evolution that we also use for the least squares optimization and take the following assumptions:

- The level of saturation *C* lays between the last value in the historical time series that we use for curve fitting and the potential limit and *C* has the same unit as the variable that is described by the S-curve, e.g. kW for the variable of solar PV capacity.
- The position of the inflection point t_0 given in years lays within this century, i.e. between the years 2000 and 2100, for logistic, Gompertz, and Bass models, and between 1900 and 2100 for Bertalanffy and the two versions of the generalized Richards model.
- The unitless degree of a function *d* is limited to a maximum of ten to reduce computational complexity.
- The vertical shift *z* lays between zero and the first value in the historical time series that was used for curve fitting and has the same unit as *C*.
- The unitless curve parameters *b, k, p, q* lay within zero and one to reduce computational complexity.

For the bi-S-curve models, we use an additional constraint so that the level of saturation *C* of the second growth phase must be higher or equal to the saturation level of the first growth phase.

A.7 Comparison of national projections from modeling on national data and from aggregating municipality-level probabilistic projections

Here, we compare the accuracy of the national projections created in two ways: (i) by aggregating the probabilistic projections of all municipalities as demonstrated in the main manuscript, and (ii) national projections that we create by fitting the twelve S-curve models to the national historical time series data of each technology. We measure the accuracy in terms of MAPE and perform iterative hindcasting as described in Table S1d. While the municipality-based national projections are probabilistic, the national projections of the S-curve models are deterministic as our modeling approach requires a data set of multiple historical time series at national level that can be grouped to create probabilities (see Table S1c). To get a more comprehensive overview, we show the results for all twelve S-curve models investigated in this study. In the extreme case, a projection based on weighted models can be the same as the projection of the best performing model.

While the accuracies of the national projections from aggregated municipalities and from the twelve national-level S-curves are comparable for heat pumps and BEVs, the evolution of the MAPE for the municipality-based projection of solar PV remains notably lower over the projected years (Figure S3). Only the two Bertalanffy models perform similar than the municipality-based projection. Considering our main finding that probabilistic projections show on average higher accuracy than the corresponding deterministic projections, the accuracy of projections at the national level might improve further. Therefore, for cases where only a projection at national level is of interest, modeling approaches that create projections by fitting models to national data may be sufficient. However, such national projections would show two major limitations. First, they require alternative ways than ours to create probabilistic density intervals, for instance, using Monte Carlo sampling, that stay untested against large sets of historical data and might therefore be less reliable. Second, while consistent projections on any level between municipality and national are possible with the municipality-based approach, the projections created with national data cannot be disaggregated without further assumptions.

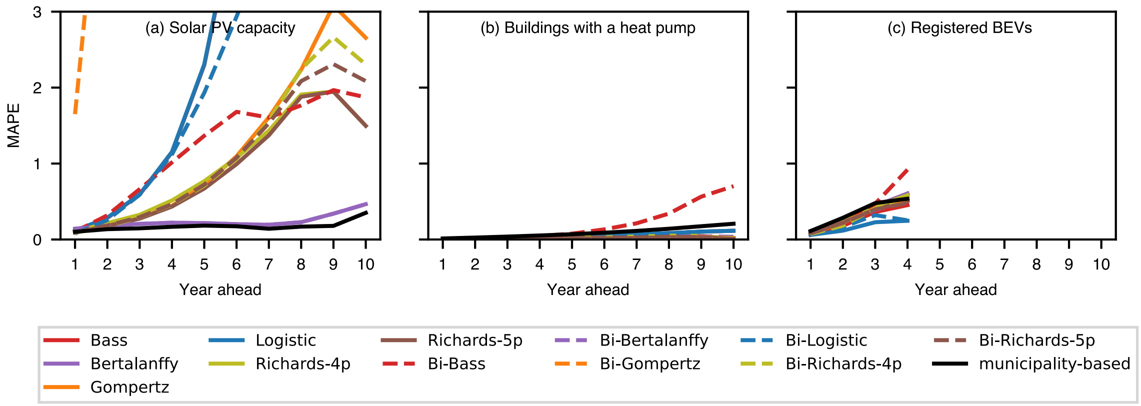

Figure S3. Temporal evolution of the mean absolute percentage error (MAPE) for deterministic projections of twelve S-curve models (colored) based on national values, and the median of aggregated municipality-level probabilistic projections (black) of the Swiss total of solar PV capacities (a), heat pumps (b), and battery electric vehicles (BEVs) (c), and iterations of hindcasting. Scores can lay outside the plot boundaries in certain years.

Appendix B

B.1 Heat maps with weights and scores of model performance from hindcasting

model	share of curves saturating below real value of 2021	MAPE (deterministic projection)	MAPE (probabilistic projection)	sharpness / WIS	calibration / WIS	WIS	weight	share of curves saturating below real value of 2021	MAPE (deterministic projection)	MAPE (probabilistic projection)	sharpness / WIS	calibration / WIS	WIS	weight	share of curves saturating below real value of 2021	MAPE (deterministic projection)	MAPE (probabilistic projection)	sharpness / WIS	calibration / WIS	WIS	weight
Bass	0.63	3.28	0.84	0.28	0.71	2.88	5.36	0.58	0.22	0.12	0.26	0.73	0.34	10.57	0.31	0.80	0.32	0.44	0.55	1.12	13.85
Bertalanffy	0.05	0.44	0.36	0.07	0.93	1.47	17.45	0.34	0.16	0.09	0.33	0.66	0.29	15.69	0.28	0.52	0.31	0.25	0.74	1.08	22.24
Gompertz	0.55	1.81	0.60	0.32	0.68	1.95	8.40	0.49	0.18	0.10	0.32	0.67	0.31	12.72	0.31	3.58	0.33	0.66	0.32	2.13	5.83
Logistic	0.64	3.39	0.82	0.28	0.71	2.85	5.45	0.59	0.23	0.12	0.27	0.72	0.34	10.53	0.33	0.88	0.36	0.42	0.57	1.20	12.35
Richards-4p	0.10	0.92	0.56	0.13	0.87	2.01	8.19	0.35	0.18	0.09	0.35	0.64	0.29	15.42	0.29	1.01	0.31	1.00	0.00	> 10	10.42
Richards-5p	0.12	0.92	0.54	0.14	0.86	1.95	8.34	0.35	0.18	0.09	0.35	0.65	0.29	15.32	0.27	0.98	0.33	1.00	0.00	> 10	11.10
Bi-Bass	0.50	3.11	0.85	0.37	0.62	2.89	4.29	0.61	0.26	0.11	0.44	0.55	0.34	8.87	0.27	1.49	0.38	0.73	0.26	1.62	7.43
Bi-Bertalanffy	0.05	0.44	0.36	0.07	0.92	1.49	17.07	0.31	0.18	0.09	1.00	0.00	> 10	0.01	0.18	0.57	0.30	1.00	0.00	> 10	0.24
Bi-Gompertz	0.36	4.09	0.58	0.67	0.32	2.75	5.34	0.35	0.59	0.09	0.62	0.37	0.39	8.02	0.21	4.09	0.33	0.84	0.15	2.93	2.81
Bi-Logistic	0.44	3.95	0.88	0.36	0.62	3.01	4.49	0.45	0.70	0.10	1.00	0.00	> 10	2.15	0.27	0.87	0.37	0.51	0.48	1.22	11.01
Bi-Richards-4p	0.13	1.07	0.56	0.17	0.83	1.95	7.72	0.35	0.23	0.09	1.00	0.00	> 10	0.68	0.12	0.89	0.30	1.00	0.00	> 10	1.48
Bi-Richards-5p	0.07	1.13	0.57	0.17	0.82	1.98	7.85	0.37	0.23	0.09	1.00	0.00	> 10	0.00	0.15	0.98	0.30	1.00	0.00	> 10	1.26
	Solar PV capacity per 100 inhabitants							Buildings with a heat pump per 100 inhabitants							Registered BEVs per 100 inhabitants						

Figure S4. Heat map with weights and scores of model performance from hindcasting for solar PV capacity, heat pumps, and battery electric vehicles (BEVs) per 100 inhabitants. For each column, colors rank each score from highest to lowest and vice versa for the weight. The shown values are means over all municipalities and hindcasting iterations with 1- to 10-year ahead projections for solar PV and heat pumps, and 1- to 4-year ahead projections for BEVs. For temporal evolutions, see Figures S10-S14. The mean absolute percentage error (MAPE) of a probabilistic projection quantifies the error between the median value of the projection and the real value. To enhance comparability as some bi-S-curves have scores that are multiple orders higher than 10, the highest 2% of MAPE scores, sharpness, calibration, and weighted interval scores (WIS) are removed for all models before taking the mean. Models that still have mean scores above 10 are indicated.

model	share of curves saturating below real value of 2021	MAPE (deterministic projection)	MAPE (probabilistic projection)	sharpness / WIS	calibration / WIS	WIS	weight	share of curves saturating below real value of 2021	MAPE (deterministic projection)	MAPE (probabilistic projection)	sharpness / WIS	calibration / WIS	WIS	weight	share of curves saturating below real value of 2021	MAPE (deterministic projection)	MAPE (probabilistic projection)	sharpness / WIS	calibration / WIS	WIS	weight
Bass	0.62	3.43	0.89	0.31	0.67	3.00	5.21	0.50	0.23	0.12	0.29	0.70	0.34	9.43	0.31	0.78	0.33	0.38	0.61	1.11	13.50
Bertalanffy	0.04	0.43	0.36	0.06	0.93	1.45	17.36	0.25	0.16	0.09	0.35	0.65	0.29	15.31	0.28	0.52	0.32	0.24	0.76	1.09	18.69
Gompertz	0.54	1.84	0.60	0.36	0.64	1.92	8.79	0.39	0.19	0.10	0.35	0.64	0.30	12.04	0.31	3.61	0.33	0.59	0.39	1.91	6.03
Logistic	0.63	3.48	0.88	0.31	0.68	2.99	5.20	0.52	0.24	0.12	0.29	0.70	0.34	9.41	0.33	0.88	0.33	0.37	0.62	1.15	12.52
Richards-4p	0.09	0.90	0.55	0.13	0.87	1.97	8.54	0.27	0.18	0.09	0.37	0.62	0.28	14.95	0.30	0.96	0.33	1.00	0.00	> 10	8.98
Richards-5p	0.11	0.89	0.53	0.14	0.86	1.91	8.68	0.27	0.18	0.09	0.36	0.63	0.29	14.82	0.26	1.02	0.33	0.38	0.61	1.24	11.00
Bi-Bass	0.49	3.31	0.89	1.00	0.00	> 10	3.78	0.54	0.28	0.12	0.44	0.55	0.35	8.18	0.27	1.50	0.34	0.72	0.27	1.52	7.44
Bi-Bertalanffy	0.04	0.43	0.36	1.00	0.00	> 10	16.73	0.23	0.18	0.09	1.00	0.00	> 10	1.47	0.18	0.56	0.31	1.00	0.00	> 10	1.34
Bi-Gompertz	0.36	4.32	0.58	0.72	0.27	3.03	4.82	0.28	0.57	0.09	0.62	0.37	0.37	8.16	0.21	4.26	0.32	0.80	0.19	2.49	3.57
Bi-Logistic	0.44	4.04	0.94	0.39	0.59	3.18	4.27	0.39	0.75	0.10	1.00	0.00	> 10	4.02	0.28	0.87	0.33	1.00	0.00	> 10	11.02
Bi-Richards-4p	0.13	1.04	0.54	0.17	0.82	1.89	8.15	0.28	0.24	0.09	1.00	0.00	> 10	1.59	0.13	0.88	0.31	1.00	0.00	> 10	1.45
Bi-Richards-5p	0.08	1.12	0.55	0.18	0.81	1.90	8.37	0.30	0.24	0.09	1.00	0.00	> 10	0.60	0.15	0.95	0.31	1.00	0.00	> 10	4.41
	Solar PV capacity per technical potential							Buildings with a heat pump per registered buildings							Registered BEVs per total civil passenger cars						

Figure S5. Heat map with weights and scores of model performance from hindcasting for solar PV capacity, heat pumps, and battery electric vehicles (BEVs) per potential. For each column, colors rank each score from highest to lowest and vice versa for the weight. The shown values are means over all municipalities and hindcasting iterations with 1- to 10-year ahead projections for solar PV and heat pumps, and 1- to 4-year ahead projections for BEVs. For temporal evolutions, see Figures S10-S14. The mean absolute percentage error (MAPE) of a probabilistic projection quantifies the error between the median value of the projection and the real value. To enhance comparability as some bi-S-curves have scores that are multiple orders higher than 10, the highest 2% of MAPE scores, sharpness, calibration, and weighted interval scores (WIS) are removed for all models before taking the mean. Models that still have mean scores above 10 are indicated.

B.2 Distribution of weights for probabilistic projections of solar PV, heat pumps, and BEVs

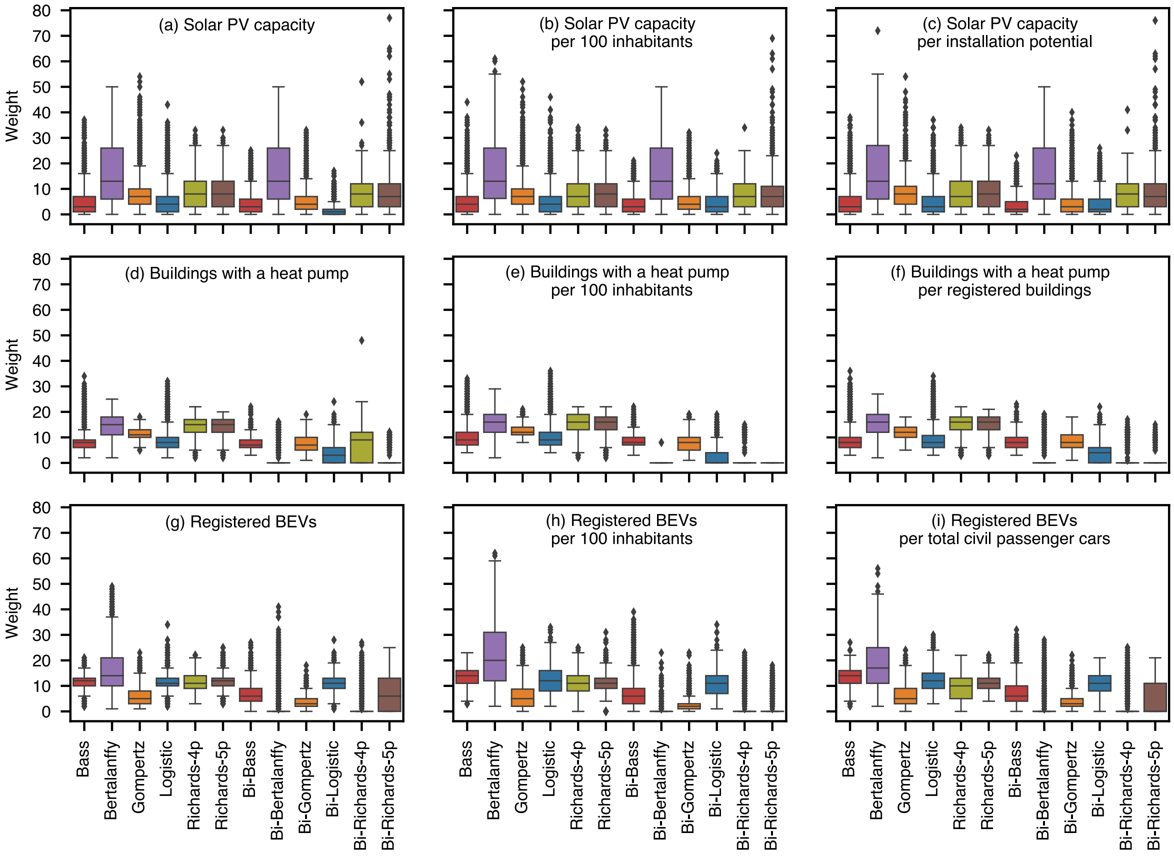

Figure S6. Box plots showing the distribution of weights for the probabilistic projections of solar PV capacities (a-c), heat pumps (d-f), and battery electric vehicles (BEVs) (g-i) across Swiss municipalities.

B.3 Diffusion of solar PV, heat pumps, and BEVs across Switzerland in 2021

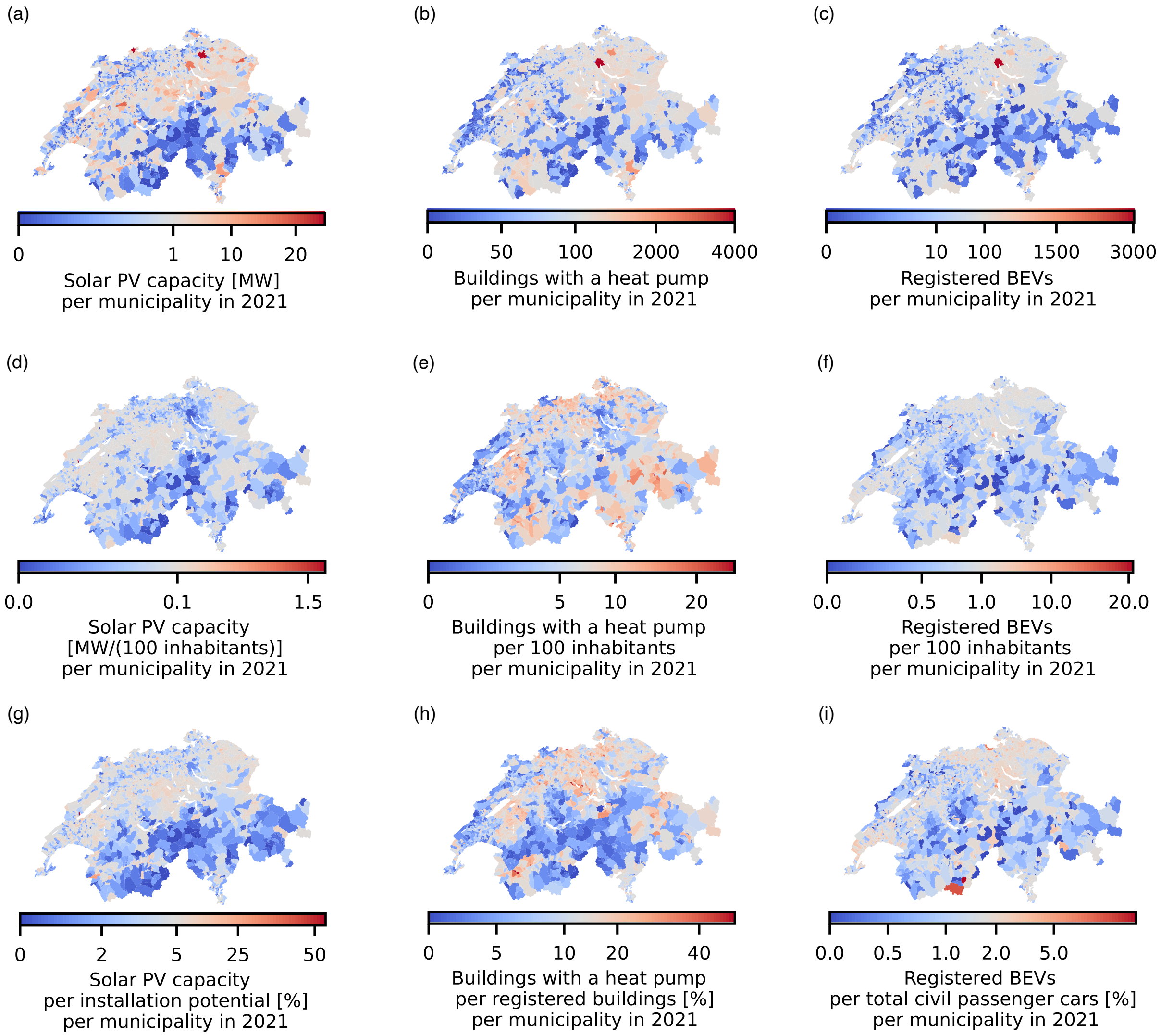

Figure S7. Distribution of solar PV capacities, heat pumps, and battery electric vehicles (BEVs) in total (a-c), per 100 inhabitants (d-f), and per potential (g-i) across Switzerland in 2021 with a quantile coloring scheme. Own visualization based on data from Swiss Federal Office of Energy and Federal Statistical Office (1, 13, 21).

B.4 Diffusion of solar PV, heat pumps, and BEVs across Switzerland in 2050

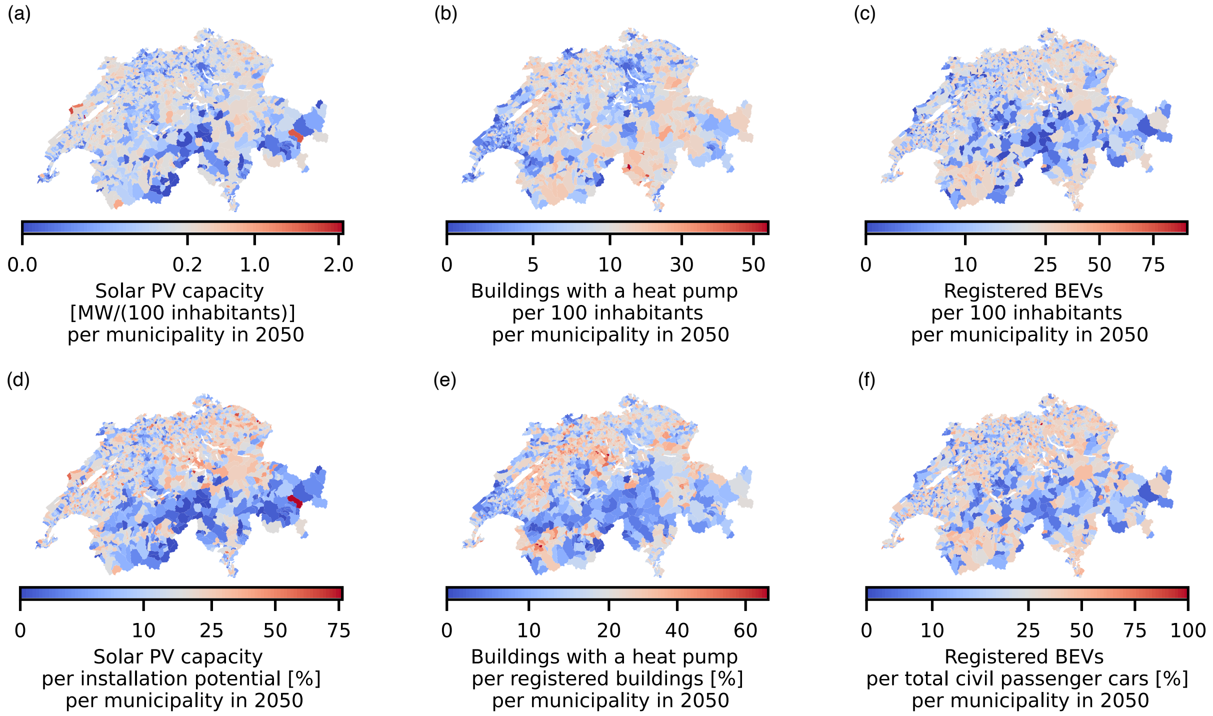

Figure S8. Distribution of solar PV capacities, heat pumps, and battery electric vehicles (BEVs) per 100 inhabitants (a-c), and per potential (d-f) across Switzerland in 2050 according to the projected median values of the probabilistic projections of each municipality and a quantile coloring scheme.

B.5 Probabilistic projections of the diffusion of solar PV and BEVs in a municipality

The probabilistic projections of solar PV, heat pumps and BEVs for all 2,148 Swiss municipalities are provided on Zenodo (22). Using the projected values, we showcase the probabilistic projections for the municipality of Thun with estimated targets for reaching net-zero greenhouse gas emissions (Figure S9). We randomly picked Thun from a group of municipalities that have a quantified energy strategy for reaching net-zero. The same could be repeated for other municipalities.

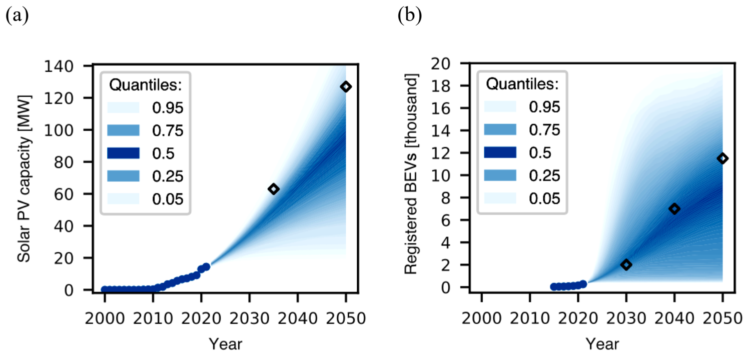

Figure S9. Probabilistic projections of the diffusion of solar PV (a) and battery electric vehicles (BEVs) (b) in the municipality of Thun until 2050. The markers (◊) set targets for reaching net-zero greenhouse gas emissions in the municipality by 2050 (23).

B.6 Temporal evolution of the mean absolute percentage error for probabilistic and deterministic projections of solar PV, heat pumps, and BEVs

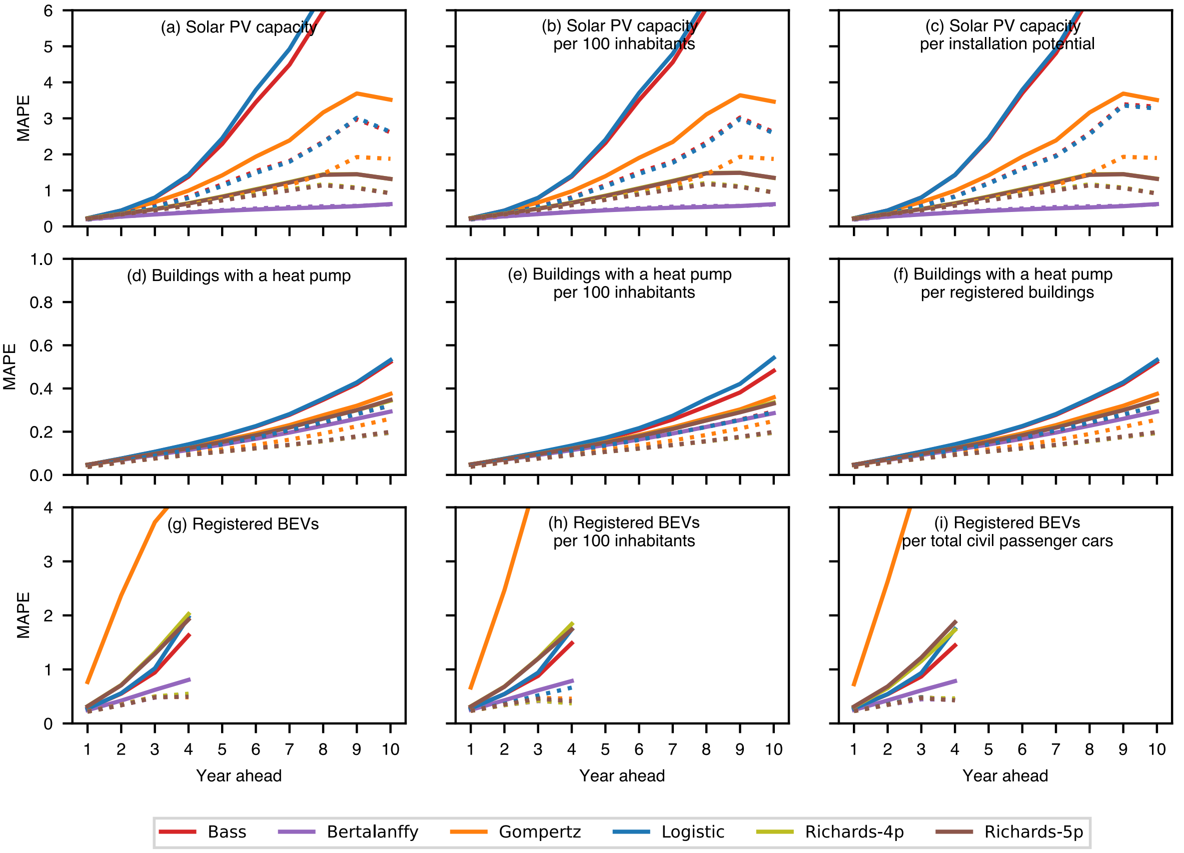

Figure S10. Temporal evolution of the mean absolute percentage error (MAPE) for the deterministic projections (solid lines) and probabilistic projections (dotted lines) of solar PV capacities (a-c), heat pumps (d-f), and battery electric vehicles (BEVs) (g-i) across Swiss municipalities and iterations of hindcasting. For visual clarity, this figure shows only uniform S-curve models whereas Figure S11 shows bi-S-curve models. To enhance comparability as some bi-S-curves have scores that are multiple orders higher than 10, the highest 2% of scores are removed for all models before taking the mean. Scores can lay outside the plot boundaries in certain years.

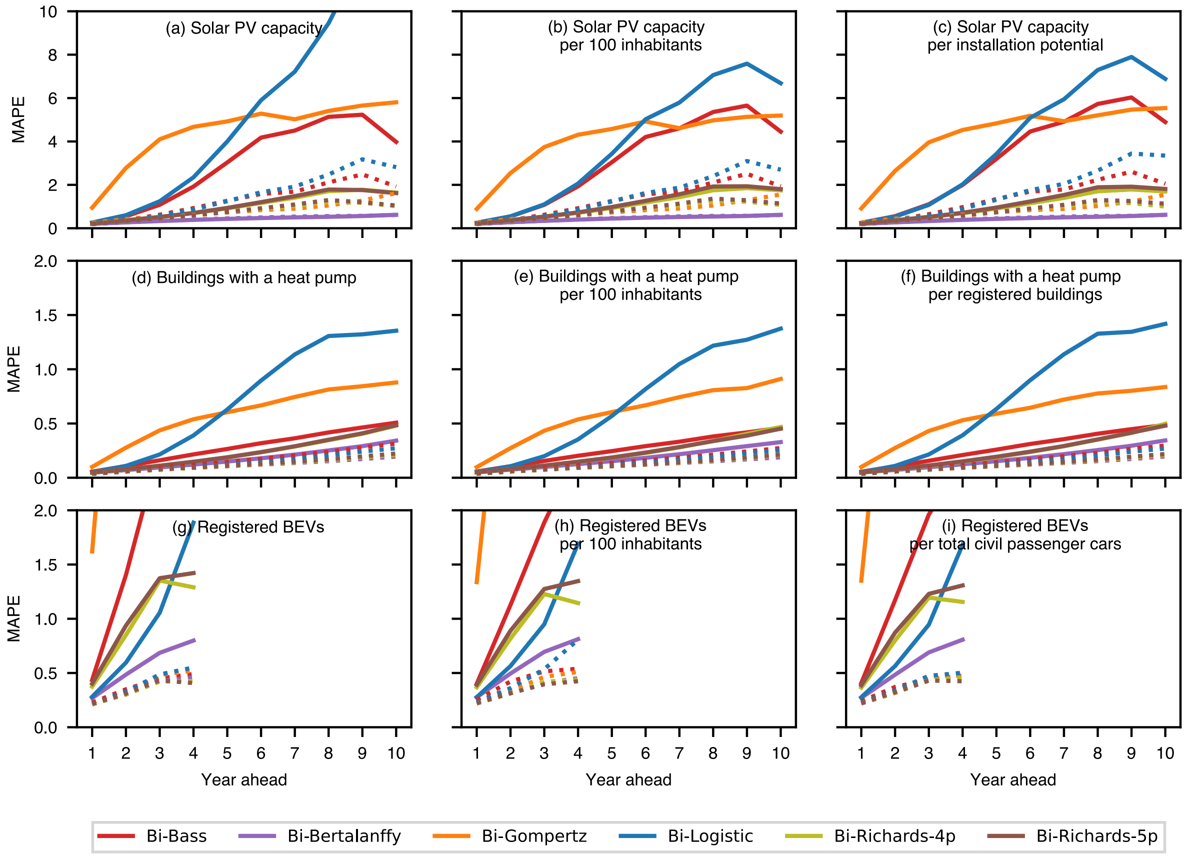

Figure S11. Temporal evolution of the mean absolute percentage error (MAPE) for the deterministic projections (solid lines) and probabilistic projections (dotted lines) of solar PV capacities (a-c), heat pumps (d-f), and battery electric vehicles (BEVs) (g-i) across Swiss municipalities and iterations of hindcasting. For visual clarity, this figure shows only bi-S-curve models whereas Figure S10 shows uniform S-curve models. To enhance comparability as some bi-S-curves have scores that are multiple orders higher than 10, the highest 2% of scores are removed for all models before taking the mean. Scores can lay outside the plot boundaries in certain years.

B.7 Temporal evolution of sharpness, calibration, and weighted interval score for probabilistic and deterministic projections of solar PV, heat pumps, and BEVs

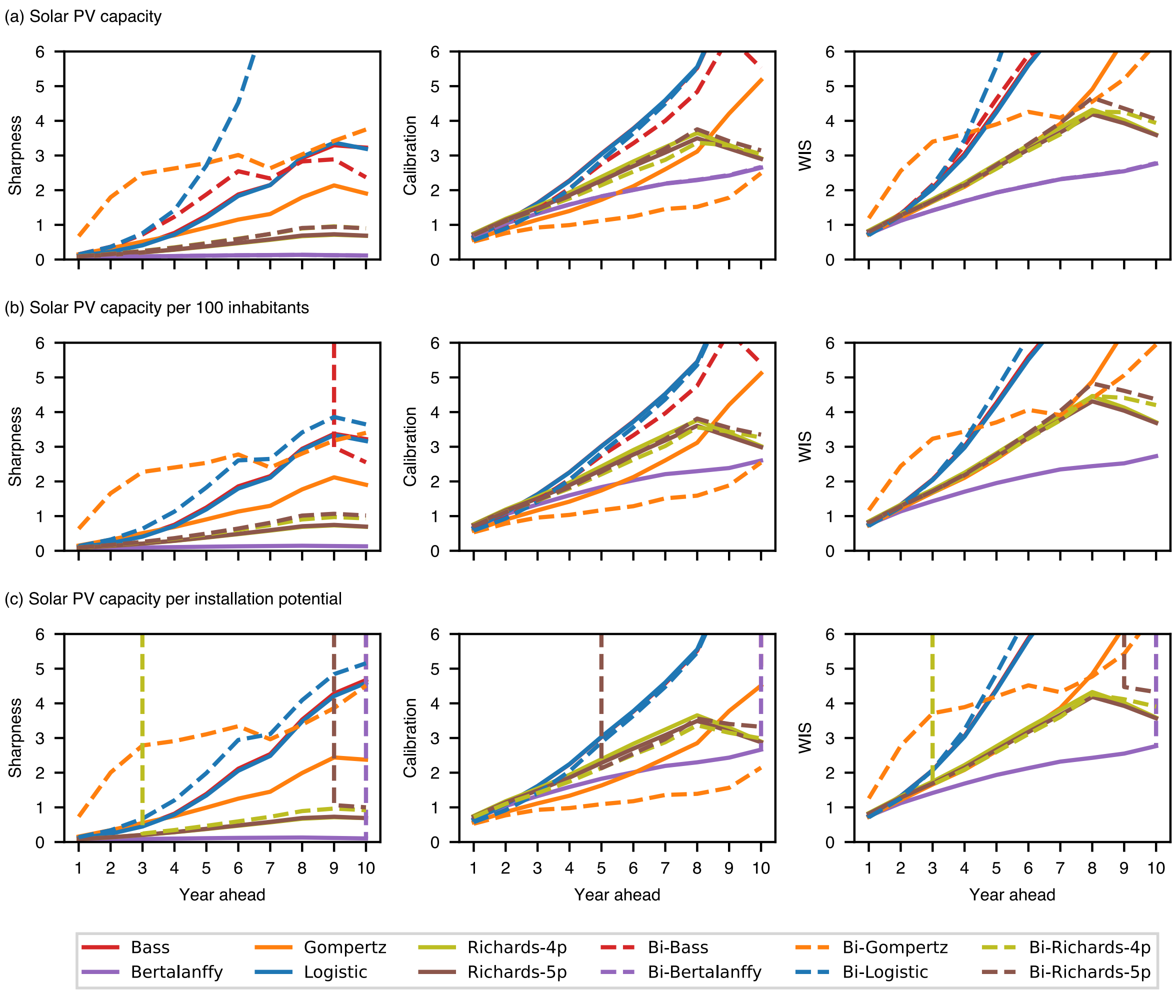

Figure S12. Temporal evolution of mean sharpness, calibration, and weighted interval score (WIS) for the probabilistic projections of solar PV capacities across Swiss municipalities and iterations of hindcasting. Scores can lay outside the plot boundaries in certain years.

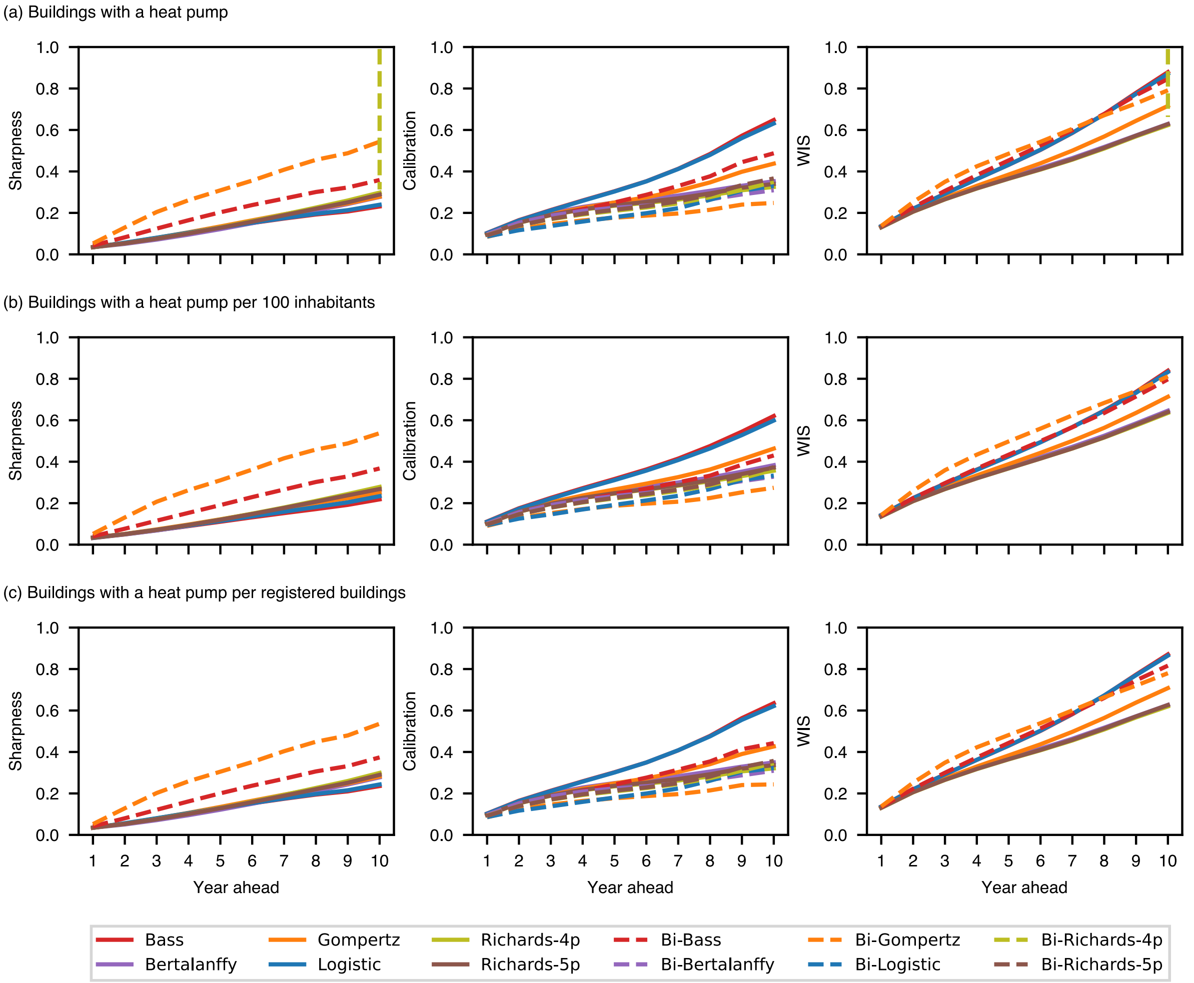

Figure S13. Temporal evolution of mean sharpness, calibration, and weighted interval score (WIS) for the probabilistic projections of buildings with a heat pump across Swiss municipalities and iterations of hindcasting. Scores can lay outside the plot boundaries in certain years.

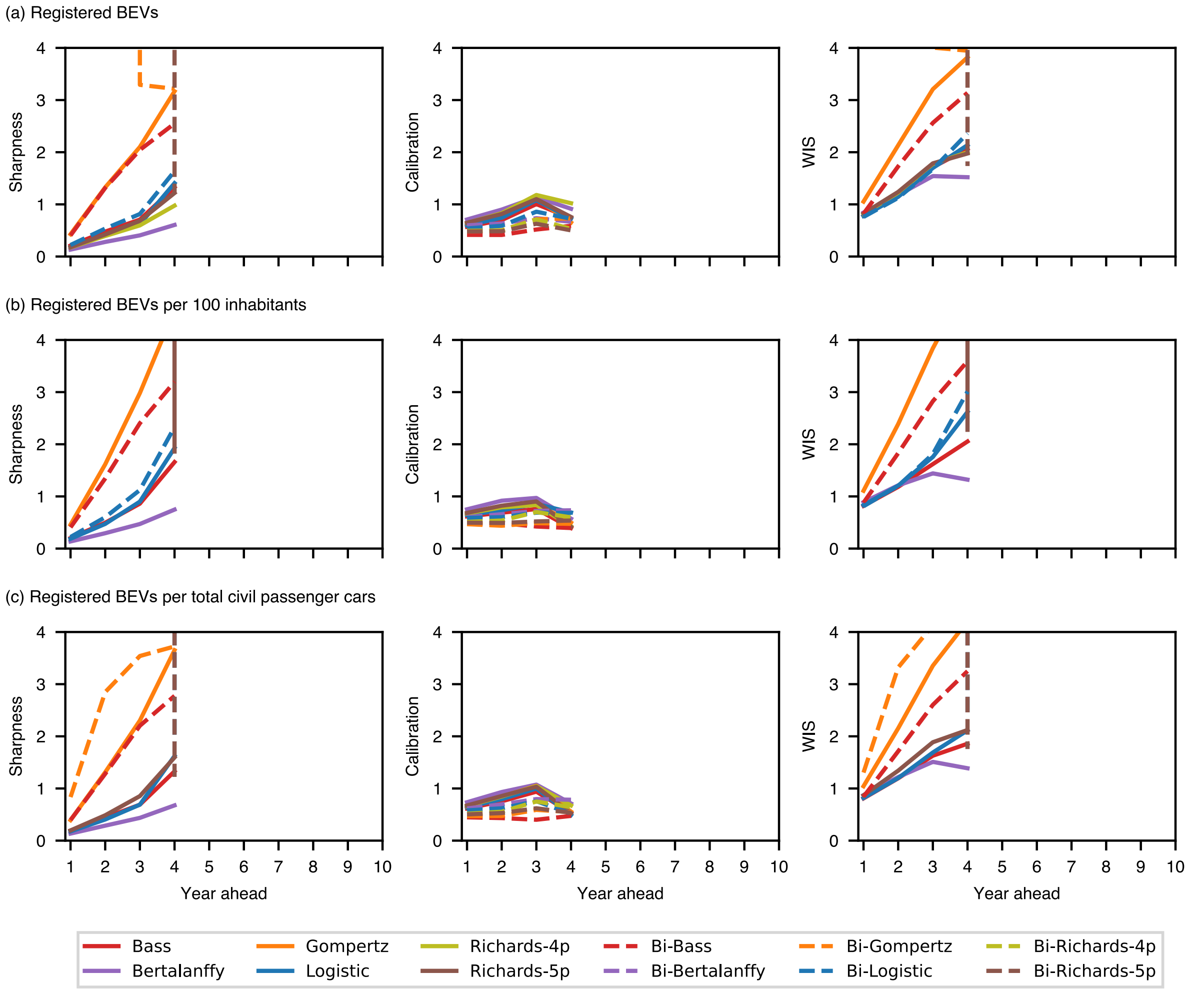

Figure S14. Temporal evolution of mean sharpness, calibration, and weighted interval score (WIS) for the probabilistic projections of battery electric vehicles (BEVs) across Swiss municipalities and iterations of hindcasting. Scores can lay outside the plot boundaries in certain years.

References

1. Federal Statistical Office (FSO), Data from "Swiss Federal Register of Buildings and Dwellings (RBD)". Available at https://www.housing-stat.ch/de/madd/index.html. Deposited 13 July 2022.

2. Federal Statistical Office (FSO), Buildings and Dwellings statistic (since 2009). Available at https://www.bfs.admin.ch/bfs/en/home/statistics/construction-housing/surveys/gws2009.html. Accessed 6 March 2023.

3. Federal Statistical Office (FSO), Buildings and Dwellings Statistics 2021. Available at https://www.bfs.admin.ch/bfs/en/home/news/whats-new.gnpdetail.2022-0075.html. Accessed 6 March 2023.

4. Federal Statistical Office (FSO), Energiebereich - Heizsystem und Energiequelle. Available at https://www.bfs.admin.ch/bfs/de/home/statistiken/bau-wohnungswesen/gebaeude/energiebereich.html. Accessed 24 October 2022.

5. geoimpact AG, WWF Schweiz, EnergieSchweiz, Data from "Energie Reporter". Available at https://opendata.swiss/de/dataset/energie-reporter. Deposited 18 January 2022.

6. P. Sterchele, *et al.*, "Studie: Wege zu einem klimaneutralen Energiesystem - Die deutsche Energiewende im Kontext gesellschaftlicher Verhaltensweisen" (Fraunhofer Institute for Solar Energy Systems ISE, Freiburg, 2020).

7. Swiss Federal Office of Energy (SFOE), Data from "Schweizerische Elektrizitätsstatistik 2021". Available at https://www.bfe.admin.ch/ogd62. Deposited 20 June 2022.

8. Basics AG, "Neue Elektro-Wärmepumpenstatistik. Dokumentation 2000." (Swiss Federal Office of Energy, Bern, 2000).

9. S. Pfenninger, I. Staffell, Long-term patterns of European PV output using 30 years of validated hourly reanalysis and satellite data. *Energy* **114**, 1251–1265 (2016).

10. S. Pfenninger, I. Staffel, Data from "Renewables.ninja". Available at https://www.renewables.ninja. Accessed 24 May 2022.

11. Federal Statistical Office (FSO), Data from "Generalisierte Gemeindegrenzen: Geodaten". Available at https://www.bfs.admin.ch/bfs/en/home/services/geostat/swiss-federal-statistics-geodata/administrative-boundaries/generalized-boundaries-local-regional-authorities.assetdetail.22484210.html. Deposited 2 May 2022.

12. Swiss Federal Office of Energy (SFOE), Data from "Solarenergiepotenziale der Schweizer Gemeinden". Available at https://opendata.swiss/de/dataset/solarenergiepotenziale-der-schweizer-gemeinden/resource/079a8be9-3c45-41fc-9ffc-80cff94cc64f. Deposited 1 January 2021.

13. Swiss Federal Office of Energy (SFOE), Data from "Elektrizitätsproduktionsanlagen". Available at https://opendata.swiss/de/dataset/elektrizitatsproduktionsanlagen. Deposited 22 June 2022.

14. Swiss Federal Office of Energy (SFOE), Swiss Solar Energy Professionals Association (Swissolar), Data from "Schweizerische Statistik der erneuerbaren Energien 2021".

Available at https://www.bfe.admin.ch/bfe/en/home/supply/renewable-energy/solar-energy.html. Deposited 1 October 2022.

15. C. Wilson, A. Grubler, N. Bauer, V. Krey, K. Riahi, Future capacity growth of energy technologies: are scenarios consistent with historical evidence? *Clim. Change* **118**, 381–395 (2013).

16. A. Debecker, T. Modis, Determination of the Uncertainties in S-Curve Logistic Fits. *Technol. Forecast. Soc. Change* **46**, 153–173 (1994).

17. A. Cherp, V. Vinichenko, J. Tosun, J. A. Gordon, J. Jewell, National growth dynamics of wind and solar power compared to the growth required for global climate targets. *Nat. Energy* **6**, 742–754 (2021).

18. Bayerische Motoren Werke Aktiengesellschaft, Impressum. Available at https://www.bmw.ch/de/footer/metanavigation/impressum/impressum-allgemein.html. Accessed 19 August 2022.

19. R. Storn, K. Price, Differential Evolution – A Simple and Efficient Heuristic for Global Optimization over Continuous Spaces. *J. Glob. Optim.*, 341–359 (1997).

20. J. Phillips, Raman Spectroscopy Fit. Available at https://bitbucket.org/zunzuncode/ramanspectroscopyfit/src/master/. Deposited 28 July 2018. *Bitbucket*.

21. Federal Statistical Office (FSO), Federal Roads Office (FEDRO), Data from “Bestand der Elektrofahrzeuge”. Available at https://www.atlas.bfs.admin.ch/maps/13/de/16504_15115_164_3114/25801.html. Deposited 27 January 2022.

22. N. Zielonka, X. Wen, E. Trutnevyte, Data from “Probabilistic projections of granular energy technology diffusion at subnational level - solar photovoltaics, heat pumps, and battery electric vehicles in Switzerland [Data set]”. Available at https://doi.org/10.5281/zenodo.8414845. Deposited 6 October 2023.

23. EBP Schweiz AG, “Grundlagen für die Klima- und Energiestrategie der Stadt Thun.” (Stadt Thun, Thun, 2021).
